# Lymphoma-associated hemophagocytic lymphohistiocytosis (LA-HLH): a scoping review unveils clinical and diagnostic patterns of a lymphoma subgroup with poor prognosis

**DOI:** 10.1038/s41375-024-02135-8

**Published:** 2024-01-18

**Authors:** Johanna Knauft, Thomas Schenk, Thomas Ernst, Ulf Schnetzke, Andreas Hochhaus, Paul La Rosée, Sebastian Birndt

**Affiliations:** 1 Klinik für Innere Medizin II, Hämatologie und internistische Onkologie, Universitätsklinikum Jena, Jena, Germany; 2 Klinik für Innere Medizin II, Onkologie, Hämatologie, Immunologie, Infektiologie und Palliativmedizin, Schwarzwald-Baar Klinikum, Villingen-Schwenningen, Germany

**Keywords:** Lymphoma, Inflammatory diseases

## Abstract

Hemophagocytic lymphohistiocytosis (HLH) is a severe hyperinflammatory syndrome driven by pathologic activation of cytotoxic T-lymphocytes and macrophages. Despite advances in diagnostics and management, adult patients with lymphoma-associated HLH (LA-HLH) harbor particularly poor prognosis and optimal treatment remains challenging. As systematic data on LA-HLH are scarce, we aimed to synthesize research evidence by thorough analysis of the published literature in PubMed (MEDLINE-database) within the context of a scoping review. Of 595 search results, 132 articles providing information on 542 patients were reviewed and analyzed. Median patient age was 60 years (range, 18–98) with male predominance (62.7%). B- and T-NHL were equally represented (45.6% and 45.2%), Hodgkin’s lymphoma was reported in 8.9% of the cases. The majority of patients (91.6%) presented in Ann-Arbor-Stages III and IV, and bone marrow infiltration was observed in a significant proportion of patients (61.5%). Soluble CD25 levels were markedly elevated (median 10,000 U/ml), with levels beyond 10,000 U/ml indicating unfavorable prognosis for 30-day and overall survival. 66.8% of the patients died after median 5.1 months. LA-HLH remains a clinical challenge requiring specialized management. Timely diagnosis and appropriate lymphoma-specific treatment are of utmost importance to enhance patient outcomes.

## Introduction

Hemophagocytic lymphohistiocytosis (HLH) is a severe hyperinflammatory syndrome driven by pathologic activation of cytotoxic T-lymphocytes and macrophages ultimately leading to cytokine storm and organ damage if not treated appropriately. First described in 1939 by Bodley Scott and Robb-Smith as histiocytic medullary reticulosis, HLH is now classified in the “H”-group of histiocytoses according to the Histiocyte Society [1, 2]. In the setting of autoimmune/autoinflammatory diseases, HLH is also referred to as macrophage activation syndrome (MAS or MAS-HLH).

Considering pathophysiology, HLH can be divided in a primary, genetically determined, and a secondary, acquired form. In primary HLH (also referred to as familial HLH, FHL), mutations in genes affecting lymphocyte toxicity or immune regulation prompt defective killing of antigen-presenting cells, thus resulting in persistent immune stimulation and overwhelming immune response. In secondary HLH, an interplay between preexisting immunosuppression, inflammation (i.e., autoinflammatory or rheumatic disorders), cytokine release within infections or malignant diseases as well as potential genetic predisposition reflects the complex pathophysiology leading to the clinical picture of hyperinflammation [3].

In contrast to primary HLH, which typically appears in infancy, secondary or acquired HLH more frequently occurs in adults. While in FHL, especially infections of viral origin represent the main trigger, the spectrum of underlying diseases in secondary HLH is diverse, with infections and malignancies being most common [4]. Of note, malignancy-associated HLH (M-HLH) may appear in the context of initial diagnosis reflecting paraneoplasia, during the disease course in case of progression or relapse, or during treatment as infection-triggered HLH. In addition, M-HLH may appear following cytokine release and T-cell activation within the context of novel therapies both in hematological and solid cancers, making the differentiation of treatment toxicity and malignancy-associated inflammation a particular challenge. As with other malignancies, lymphoma treatment increasingly incorporates immune-activating and cellular therapies (i.e., checkpoint inhibitors, T-cell engaging bispecific antibodies, chimeric antigen receptor modified T-cells), in which cytokine release is a well-known side effect with the potential to induce a life-threatening HLH-like clinical picture in extreme cases (also termed as HLH-like toxicity) [5, 6]. On the contrary, the programmed cell death protein-1 (PD-1) antibody Nivolumab demonstrated efficacy in the treatment of refractory or relapsed Epstein-Barr virus (EBV)-HLH in a pilot study, most likely due to expansion of PD-1 positive T-cells and restoring the defective anti-EBV response [7].

In the M-HLH group, lymphoma represent the most common trigger and typically exhibit the poorest prognosis [8]. This might be due to often-aggressive disease courses of certain lymphoma subtypes enriched in patients with M-HLH, but also due to distinct and unusual clinical features hindering lymphoma diagnosis. In particular, initial diagnosis may be delayed because of similarities between HLH and other inflammatory conditions such as sepsis. Moreover, histological diagnosis may be hindered by tissue infiltration with activated lymphocytes and macrophages that allow tumor cells to hide behind inflammatory infiltrates [9].

Clinically, HLH patients may present with a variety of symptoms according to the underlying disease, a triad consisting of fever, splenomegaly, and cytopenia is common though. Diagnosis of HLH is currently based on the HLH-2004 criteria established by the Histiocyte Society, whereby 5 of 8 criteria have to be fulfilled for HLH diagnosis [10]. During past few years, adaptation of these criteria has been proposed especially for adult patients with secondary HLH given the non-specificity of several of the HLH-2004 criteria, such as using higher ferritin or soluble CD25 (sCD25; synonym: soluble interleukin-2 receptor chain alpha, sIL-2Rα) cut-off values to best identify patients with HLH and distinguish from other inflammatory states like sepsis. Recently, the combination of elevated sCD25 of more than 3900 U/ml and ferritin greater 1000 µg/l showed best sensitivity and specificity in diagnosing M-HLH and might also aid in identifying patients with lymphoma-associated HLH in due time [11].

Despite these advances and a growing number of publications reporting on lymphoma-associated HLH (LA-HLH) in adults, systematic or multicenter data are still scarce given the rarity of LA-HLH. Within the context of a systematic scoping review, we therefore aimed to synthesize research evidence on LA-HLH by interrogation of the published literature in order to determine current evidence on epidemiology, LA-HLH subtypes and its clinical features, as well as providing an overview on applied therapies and prognosis.

## Methods

### Search strategy

On February 27th, 2020, a comprehensive literature search of the PubMed database was performed. To identify publications on LA-HLH, the following search terms and medical subject headings were used: “hemophagocytic lymphohistiocytosis” OR “haemophagocytic lymphohistiocytosis” AND “lymphoma-associated hemophagocytic lymphohistiocytosis” OR “hemophagocytic syndrome lymphoma” OR “haemophagocytic syndrome lymphoma” OR “Hodgkin hemophagocytic lymphohistiocytosis” OR “Hodgkin haemophagocytic lymphohistiocytosis” OR “T-cell non-Hodgkin lymphoma hemophagocytic lymphohistiocytosis” OR “T-cell non-Hodgkin haemophagocytic lymphohistiocytosis” OR “diffuse large B-cell lymphoma hemophagocytic lymphohistiocytosis” OR “diffuse large B-cell lymphoma haemophagocytic lymphohistiocytosis” OR “chronic lymphocytic leukemia hemophagocytic lymphohistiocytosis” OR “chronic lymphocytic leukemia haemophagocytic lymphohistiocytosis”.

### Study selection and data collection

All case reports and studies referring to adult patients (≥18 years of age) with HLH triggered by lymphoma were screened. We considered patients to have HLH if they met at least five out of eight HLH-2004 diagnostic criteria or authors reported a diagnosis of HLH respectively. Only articles published in English language were included. We excluded articles focusing on pediatric patients, reporting insufficient data, or if lymphoma was not considered being the most likely HLH-triggering disease.

**Fig. 1:**
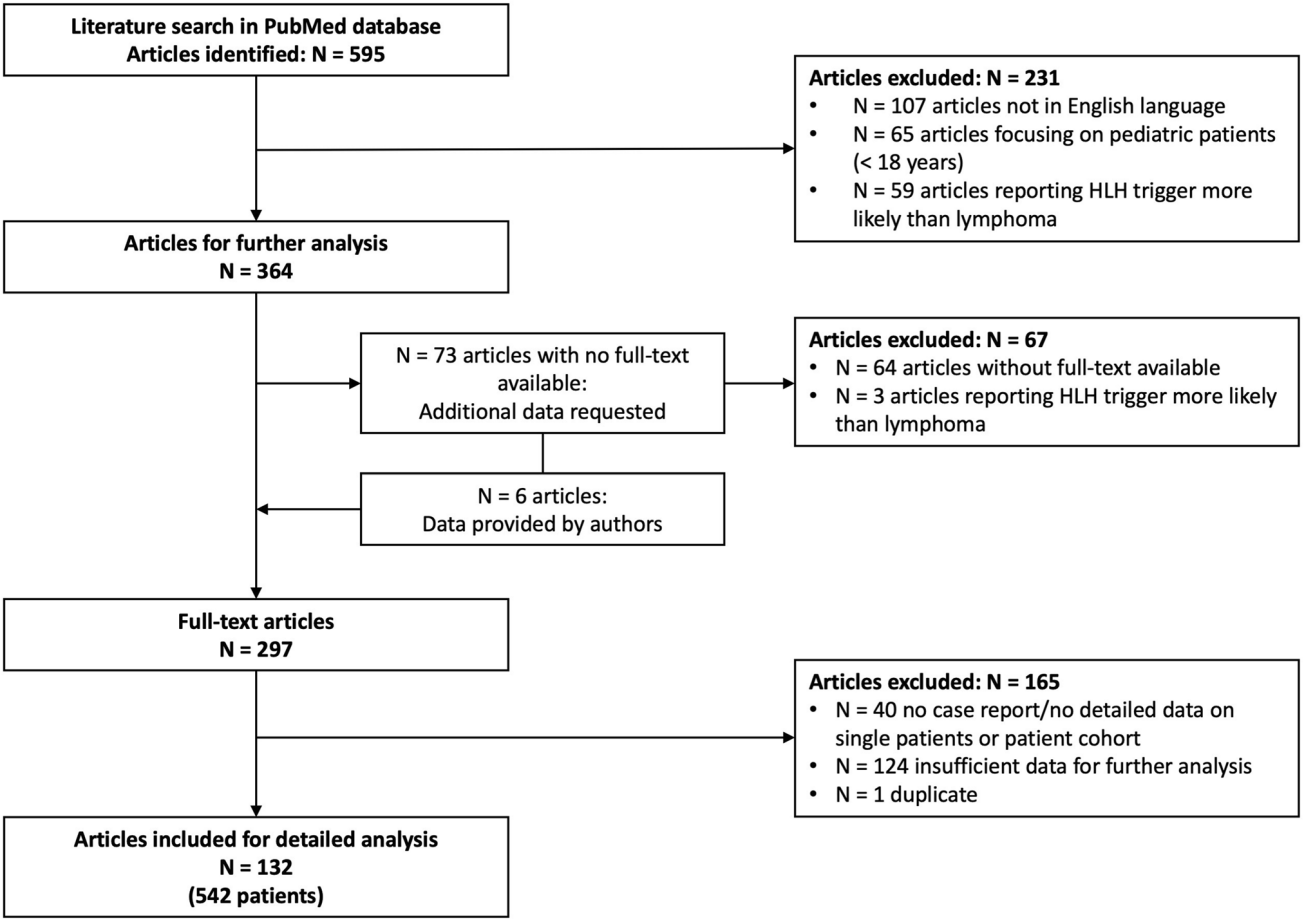
PRISMA flow diagram showing study selection process.

Study selection followed a three-step analysis process. First, based on inclusion and exclusion criteria, titles and abstracts of all 595 PubMed results were assessed for their relevance. Secondly, full-text content of articles was obtained if available. Of 73 articles with no available full text, the corresponding authors were requested to provide data. Subsequently, 67 articles were excluded from further analysis (64 articles without available full-text and three articles reporting on HLH cases not associated with lymphoma). In a third step, the remaining 297 full-text articles were reassessed using the abovementioned criteria and with consideration of data quality, resulting in 132 publications for detailed analysis. Figure 1 illustrates the process of study selection using a PRISMA flow diagram.

Data on lymphoma subtype (B-cell non-Hodgkin lymphoma, T-cell non-Hodgkin lymphoma (T-NHL), Hodgkin’s lymphoma, unspecified lymphoma), patient characteristics, clinical and laboratory parameters, treatment, and outcome were extracted depending on availability. Survival times if provided in the analyzed articles were extracted and recorded in months. In case the authors reported more than one associated lymphoma, the most likely HLH-triggering diagnosis was listed accordingly. To avoid analyzing duplicates (i.e., overlapping patient cohorts), author names and affiliations and year of publication were compared. Patient demographics were subsequently reviewed to identify cases published more than once.

### Statistical analysis

Descriptive data are presented as median and corresponding ranges or frequencies (%). We used Chi-Square test to compare differences regarding categorial variables and Mann-Whitney *U* test for continuous variables. Survival times were recorded as provided in the analyzed articles and visualized using Kaplan-Meier method, the log-rank test was performed for comparison of subgroups. Further survival analysis was carried out using Kaplan-Meier method and Cox’s proportional hazards model. Variables with a *p* < 0.05 in univariate analysis were included in multivariate analysis using Cox’s proportional hazards model with a backward stepwise selection procedure. All statistical tests were two-sided. A *p*  < 0.05 was considered statistically significant. All statistical analyses were carried out using IBM SPSS Statistics Version 27 (IBM Corp., Armonk, N.Y., US).

## Results

### Epidemiology

Of 595 search results, 132 articles (*N* = 113 case reports [12–124]; *N* = 19 single or multicenter studies [125–143]) providing data on 542 LA-HLH patients were eligible for further analysis and included in this study. Three hundred forty patients were male (62.7%) and median age was 60 years (range, 18–98 years), patients with T-NHL had a lower median age compared to those with B-cell non-Hodgkin lymphoma (B-NHL) (49 vs. 64 years, *p* < 0.001). As expected, given the growing diagnostic vigilance, the number of publications concerning LA-HLH showed a steady increase during the past years (Supplementary Fig. 1).

**Fig. 2:**
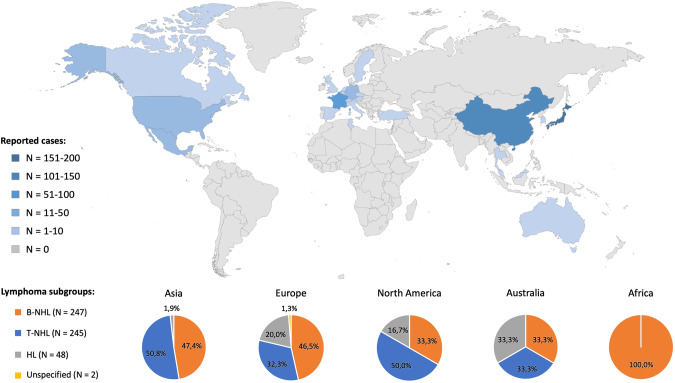
Map showing the geographic distribution of *N* = 542 reported patients and corresponding lymphoma subgroups. Abbreviations: B-NHL B-cell non-Hodgkin lymphoma, T-NHL T-cell non-Hodgkin lymphoma, HL Hodgkin’s lymphoma.

The majority of reported patients came from Asia (*N* = 323/542, 59.6%), followed by Europe and North America (28.6% and 11.1%, respectively). In more detail, most of the published patients (pts) were from Japan and China (174 and 138 pts, respectively), followed by France (93 pts). Figure 2 provides an overview on the geographic distribution and shows corresponding lymphoma subgroups, Supplementary Table 1 shows countries of origin and the corresponding lymphoma subtypes. Of note, T-NHL was reported more frequently in publications originating from China compared to all other countries, with particular emphasis on natural killer/T-cell lymphoma (NKTCL).

### Lymphoma subtypes

**Fig. 3:**
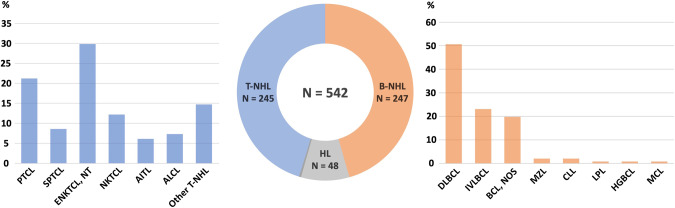
Distribution of HLH-triggering lymphoma subtypes. Two patients had HLH due to not further specified lymphoma. Abbreviations: B-NHL B-cell non-Hodgkin lymphoma, T-NHL T-cell non-Hodgkin lymphoma, HL Hodgkin’s lymphoma, PTCL, NOS Peripheral T-cell lymphoma, not otherwise specified, SPTCL Subcutaneous panniculitis-like T-cell lymphoma, ENKTCL, NT Extranodal natural killer/T-cell lymphoma, nasal type, NKTCL Natural killer/T-cell lymphoma, AITL Angioimmunoblastic T-cell lymphoma, ALCL Anaplastic large-cell lymphoma, DLBCL Diffuse large B-cell lymphoma, BCL, NOS B-cell lymphoma, not otherwise specified, MZL Marginal zone lymphoma, CLL Chronic lymphocytic leukemia, LPL Lymphoplasmacytic lymphoma, HGBCL High-grade B-cell lymphoma, MCL Mantle cell lymphoma.

There was an equal distribution of T-NHL and B-NHL-triggered HLH (45.2% and 45.6% of all patients, respectively). Hodgkin’s lymphoma-associated HLH was reported in 8.9% of all cases. The most common T-NHL subgroups according to 2016 World Health Organization classification of lymphoid neoplasms [144] were peripheral T-cell lymphoma, not otherwise specified (21.2% of all T-NHL-HLH cases) and extranodal NKTCL, nasal type (29.8%). Moreover, subcutaneous panniculitis-like T-cell lymphoma (SPTCL) and angioimmunoblastic T-NHL (AITL) were frequently reported (8.6% and 6.1%, respectively). In the B-NHL group diffuse large B-cell lymphoma represents the main etiology (50.6% of all B-NHL-associated HLH cases), followed by intravascular large B-cell lymphoma (23.1%). Figure 3 shows a graphic impression of different LA-HLH subgroups, and Supplementary Table 2 gives a detailed listing of lymphoma subtypes with their percentage distributions.

### Patient characteristics, clinical features

Besides typical symptoms like fever, or enlarged liver and spleen, there was a wide spectrum of symptoms caused by involvement of liver, lung, or the gastrointestinal tract. Of note, there was a high percentage of bone marrow infiltration due to lymphoma throughout all subgroups, which was highest in the B-NHL subgroup with almost 80%. The vast majority of patients (91.6%) presented in Ann-Arbor stages III and IV. Half of the patients with T-NHL exhibited skin changes. Table 1 comprises clinical features of the total lymphoma cohort, and patients grouped by subentities.

**Table 1:** Clinical features and organ involvement in patients with lymphoma-associated HLH.

	Total	Mixed^a^	B-NHL	T-NHL	HL
Male	340/542 (62.7)	130/189 (68.8)	105/182 (57.7)	87/147 (59.2)	18/24 (75.0)
Fever	379/395 (95.9)	118/118 (100.0)	133/142 (93.7)	104/111 (93.7)	24/24 (100.0)
Splenomegaly	390/485 (80.4)	154/189 (81.5)	132/162 (81.5)	86/110 (78.2)	18/24 (75.0)
Hepatomegaly	238/382 (62.3)	105/148 (70.9)	74/123 (56.1)	48/88 (54.5)	11/23 (47.8)
Hemophagocytosis^b^	300/368 (81.5)	83/113 (73.5)	124/136 (91.2)	75/95 (78.9)	18/24 (75.0)
Lymphadenopathy	145/310 (46.8)	50/71 (70.4)	27/107 (25.2)	51/110 (46.4)	17/22 (77.3)
*Affected organs/organ systems due to lymphoma infiltration:*					
Bone marrow	192/312 (61.5)	31/71 (43.7)	78/102 (76.5)	72/121 (59.5)	11/18 (61.1)
Lymph node	105/254 (41.3)	44/71 (62.0)	20/87 (23.0)	28/78 (35.9)	13/18 (72.2)
Liver	89/266 (33.5)	42/104 (40.4)	19/70 (27.1)	25/74 (33.8)	3/18 (16.7)
Skin	77/306 (25.2)	20/104 (19.2)	11/89 (12.4)	46/91 (50.5)	0/22 (0.0)
Spleen	65/239 (27.2)	29/71 (40.8)	14/77 (18.2)	20/73 (27.4)	2/18 (11.1)
Lung	28/217 (12.9)	8/33 (24.2)	10/88 (11.4)	6/73 (8.2)	4/23 (17.4)
Gastrointestinal tract	19/247 (7.7)	12/104 (11.5)	2/68 (2.9)	5/56 (8.9)	0/19 (0.0)
Kidney	13/174 (7.5)	6/33 (18.2)	3/67 (4.5)	4/56 (7.1)	0/18 (0.0)
Nervous system	6/174 (3.4)	2/33 (6.1)	3/67 (4.5)	1/56 (1.8)	0/18 (0.0)
*Ann Arbor stage:*					
I + II	22/263 (8.4)	4/110 (3.6)	1/57 (1.8)	15/77 (19.5)	2/19 (10.5)
III + IV	241/263 (91.6)	106/110 (96.4)	56/57 (98.2)	62/77 (80.5)	17/19 (89.5)

*B-NHL* B-cell non-Hodgkin lymphoma. *T-NHL* T-cell non-Hodgkin lymphoma, *HL* Hodgkin’s lymphoma.

Data are provided as n/N and corresponding percentages in parentheses. Respective numbers of patients depend on data availability.

^a^Indicates pooled data of LA-HLH patients, in which a precise breakdown according to lymphoma subtype was not possible.

^b^Assessed in bone marrow, spleen, or lymph node.

### Laboratory features

Single or more extensive laboratory values were available for 225 patients. About 60% of the patients presented with cytopenia of at least two lineages; thrombocytopenia less than 100 × 10^9^/l was observed most frequently (85.6%), followed by anemia and neutropenia (61.4 and 44.2%, respectively). Ferritin as a hallmark of HLH was increased in 90% of the patients, median ferritin level was 4808 µg/l (range, 12–526,259), and 30% of the patients presented with extremely elevated values beyond 15,000 µg/l. Comparing different lymphoma subgroups, patients with T-NHL showed significantly higher median ferritin levels than the B-NHL group (7604 vs. 2785 µg/l, *p* = 0.045) and more often had values of above 10,000 µg/l (47.8 vs. 23.6%, *p* = 0.003). Elevated triglycerides were observed in 62% of the patients, while low fibrinogen was present in 42%. Virtually all patients had elevated soluble CD25 (sCD25) levels, with a median of 10,380 U/ml (range, 1194–108,640) for the entire cohort. Comparing sCD25 levels between lymphoma subgroups, there was no significant difference between the B-NHL and T-NHL group (median 9585 U/ml [range, 1194–71,834] vs. 14,000 U/ml [range, 3588–108,640], *p* = 0.392). However, the sCD25/ferritin ratio was higher in patients with HLH triggered by B-NHL. Patients with Hodgkin’s lymphoma-HLH showed the highest sCD25 levels with a median level of 24,414 U/ml (range, 4250–96,415). Lactate dehydrogenase levels were elevated in nearly all patients (97.3%), with a significant increase of more than five times the upper limit of normal in one third (34.4% of the patients). Patients with T-NHL tended to have lower platelet counts and lower fibrinogen than B-NHL patients. Table 2 shows detailed laboratory features of all patients with available data and grouped into B- and T-NHL associated HLH, Supplementary Table 6 provides laboratory features of Hodgkin’s lymphoma-HLH patients.

**Table 2:** Detailed laboratory information for all LA-HLH patients with available data (*N* = 225) and divided into B- or T-NHL groups.

		Entire LA-HLH cohort		B-NHL		T-NHL	*p* value
Number of patients		225		107		94	n.a.
Male		129/225 (57.3)		59/107 (55.1)		52/94 (55.3)	1.000
Age (years)	*n* = 224	60 [18–98]	*n* = 107	64 [21–98]	*n* = 93	49 [18–78]	<0.001^a^
Anemia (Hemoglobin <90 g/l)		127/207 (61.4)		61/104 (58.7)		45/79 (57.0)	0.937
Hemoglobin (g/l)	*n* = 205	84 [37–147]	*n* = 102	87 [37–147]	*n* = 79	85 [42–137]	0.691
Leukopenia (WBC count <4 × 10^9^/l)		116/166 (69.9)		37/82 (45.1)		64/67 (95.5)	<0.001^a^
Leukocyte count (×10^9^/l)	*n* = 166	2.7 [0.1–64.1]	*n* = 82	4.3 [0.2–29.3]	*n* = 68	1.8 [0.1–64.1]	<0.001^a^
Neutropenia (ANC <1 × 10^9^/l)		38/86 (44.2)		11/33 (33.3)		21/43 (48.8)	0.262
ANC <0.5 × 10^9^/l		14/79 (17.7)		4/31 (12.9)		8/40 (20.0)	0.532
Neutrophils (×10^9^/l)	*n* = 79	1.2 [0.1–40.1]	*n* = 31	1.9 [0.1–7.9]	*n* = 40	1.0 [0.1–40.1]	0.095
Thrombocytopenia (Platelets <100 × 10^9^/l)		179/209 (85.6)		87/105 (82.9)		69/80 (86.3)	0.671
Platelets <20 × 10^9^/l		40/207 (19.3)		13/103 (12.6)		22/80 (27.5)	0.019^a^
Platelet count (×10^9^/l)	*n* = 207	47 [2–1212]	*n* = 103	58 [2–1212]	*n* = 80	40 [5–207]	0.035^a^
Cytopenia (≥2 lineages)		119/205 (58.0)		56/103 (54.4)		42/78 (53.8)	1.000
Hyperferritinemia (≥500 μg/l)		163/182 (89.6)		82/89 (92.1)		57/69 (82.6)	0.114
Ferritin >1000 μg/l		142/182 (78.0)		69/89 (77.5)		49/69 (71.0)	0.454
Ferritin >10,000 μg/l		68/182 (37.4)		21/89 (23.6)		33/69 (47.8)	0.003^a^
Ferritin >15,000 μg/l		56/182 (30.8)		16/89 (18.0)		29/69 (42.0)	0.002^a^
Ferritin (μg/l)	*n* = 182	4808 [12–526,259]	*n* = 89	2785 [41–270,458]	*n* = 69	7604 [12–526,259]	0.045^a^
Hypertriglyceridemia (≥265 mg/dl)		56/90 (62.2)		26/35 (74.3)		22/39 (56.4)	0.145
Triglycerides (mg/dl)	*n* = 90	309 [75–2257]	*n* = 35	384 [138–2257]	*n* = 39	276 [77–1256]	0.055
Hypofibrinogenemia (<150 mg/dl)		37/88 (42.0)		15/45 (33.3)		20/30 (66.7)	0.009^a^
Fibrinogen (mg/dl)	*n* = 87	162 [0–848]	*n* = 44	182 [33–848]	*n* = 30	102 [40–604]	<0.001^a^
Soluble CD25 (≥2400 U/ml)		93/95 (97.9)		62/64 (96.9)		19/19 (100.0)	1.000
sCD25 >3900 U/ml		87/93 (93.5)		57/62 (91.9)		18/19 (94.7)	1.000
sCD25 >10,000 U/ml		48/93 (51.6)		29/62 (46.8)		11/19 (57.9)	0.441
sCD25 (U/ml)	*n* = 93	10,380 [1194–108,640]	*n* = 62	9585 [1194–71,834]	*n* = 19	14,000 [3588–108,640]	0.392
sCD25/ferritin ratio	*n* = 93	2.48 [0.04–146.10]	*n* = 62	4.61 [0.04–146.10]	*n* = 19	0.91 [0.08–15.54]	0.002^a^
ALAT > ULN		97/143 (67.8)		39/70 (55.7)		43/55 (78.2)	0.009^a^
ALAT > 5 × ULN		24/117 (20.5)		9/58 (15.5)		11/44 (25.0)	0.232
ALAT > 10 × ULN		9/117 (7.7)		4/58 (6.9)		5/44 (11.4)	0.494
ASAT > ULN		95/120 (79.2)		42/54 (77.8)		40/50 (80.0)	0.782
ASAT > 5 × ULN		29/105 (27.6)		8/48 (16.7)		17/42 (40.5)	0.012^a^
ASAT > 10 × ULN		9/105 (85.7)		4/48 (8.3)		5/42 (11.9)	0.729
Hypoalbuminemia (<35 g/l)		45/46 (97.8)		24/24 (100.0)		11/12 (91.7)	0.333
Albumin (g/l)	*n* = 46	21 [11–40]	*n* = 24	22 [15–30]	*n* = 12	20 [14–40]	0.685
Total bilirubin >1.1 mg/dl		48/71 (67.6)		26/46 (56.5)		15/17 (88.2)	0.035^a^
Total bilirubin (mg/dl)	*n* = 71	2.1 [0.4–248.0]	*n* = 46	1.2 [0.4–248.0]	*n* = 17	3.3 [0.7–19.1]	0.003^a^
LDH > ULN		179/184 (97.3)		94/96 (97.9)		69/71 (97.2)	1.000
LDH > 5 × ULN		45/131 (34.4)		19/58 (32.8)		24/57 (42.1)	0.300
LDH > 10 × ULN		14/131 (10.7)		6/58 (10.3)		8/57 (14.0)	0.545

*LA-HLH* lymphoma-associated HLH, *B-NHL* B-cell non-Hodgkin lymphoma, *T-NHL* T-cell non-Hodgkin lymphoma, *WBC* white blood cell, *ANC* absolute neutrophil count, *ALAT* alanine aminotransferase, *ASAT* aspartate aminotransferase, *LDH* lactate dehydrogenase, *ULN* upper limit of normal, *sCD25* soluble CD25.

Data are presented as median and range in parentheses or n/N with corresponding percentages. Moreover, the respective number of patients with available data is indicated.

^a^Indicates statistically significant values.

EBV status was available for 131 patients. Of these, 29 (22.1%) had past infection and 49 (37.4%) presented with EBV reactivation defined as active EBV replication. Comparing lymphoma subgroups, patients with T-NHL showed a higher percentage of EBV replication than B-NHL (41.7 vs. 18.8%), while virtually all Hodgkin’s lymphoma patients exhibited EBV replication (89.5%). Data on EBV status are comprised in Supplementary Table 3.

### Treatment

Detailed information on HLH treatment and lymphoma-specific therapy (or if no such treatment was administered) was available for 184/542 patients (33.9%) and 280/542 patients (51.7%), respectively. 94.3% (264/280) of the patients with available information received treatment for either HLH, lymphoma, or both. Forty patients (14.3%) were treated for HLH only, while 222 patients (79.3%) received lymphoma-specific treatment (alone or in combination) and 90 patients (32.1%) were treated for HLH and lymphoma. Sixteen patients (5.7%) received neither HLH- nor lymphoma-specific treatment. 29 of 222 patients with information on lymphoma treatment (13.1%) received hematopoietic stem cell transplantation (HSCT) (allogeneic or autologous). Table 3 provides an overview on HLH and lymphoma-specific treatment strategies in more detail.

**Table 3:** HLH and lymphoma-specific treatment strategies in patients with LA-HLH.

	Total	B-NHL	T-NHL	HL
Number of patients	184	76	87	21
No HLH-directed therapy	50 (27.2)	24 (31.6)	23 (26.4)	3 (14.3)
HLH-directed therapy	134 (72.8)	52 (68.4)	64 (73.6)	18 (85.7)
CS monotherapy	45 (33.6)	26 (50.0)	12 (18.8)	7 (38.9)
CS in combination	132 (98.5)	51 (98.1)	63 (98.4)	18 (100.0)
Modified HLH-94 protocol	53 (39.6)	15 (28.8)	28 (43.8)	10 (55.6)
Etoposide^a^	56 (41.8)	15 (28.8)	31 (48.4)	10 (55.6)
Cyclophosphamide^a^	13 (9.7)	5 (9.6)	7 (10.9)	1 (5.6)
Vinca alkaloids^a^	15 (11.2)	1 (1.9)	14 (21.9)	0
Cyclosporine A^a^	24 (17.9)	6 (9.1)	16 (25.0)	2 (11.1)
Antibodies^a^	20 (14.9)	9 (11.5)	5 (7.8)	6 (33.3)
Cytokine filtration^a^	1 (0.7)	0	0	1 (5.6)
Other	2 (1.5)	1 (1.9)	1 (1.6)	0
Number of patients	280	124	135	21
No Lymphoma-specific therapy	58 (20.7)	22 (17.7)	32 (23.7)	4 (19.0)
Lymphoma-specific therapy	222 (79.3)	102 (82.3)	103 (76.3)	17 (81.0)
CS monotherapy	7 (3.2)	3 (2.9)	4 (3.9)	0
CHOP/CHOP-like	121 (54.5)	70 (68.6)	49 (47.6)	2 (11.8)
CHOEP ± Rituximab	32 (14.4)	18 (17.6)	14 (13.6)	0
Rituximab^a^	54 (24.3)	50 (49.0)	1 (1.0)	3 (17.6)
Etoposide^a^	55 (24.8)	21 (19.6)	30 (30.1)	4 (23.5)
Stem cell transplantation (SCT)	29 (13.1)	14 (13.7)	13 (12.6)	2 (11.8)
Autologous SCT	21 (9.5)	14 (13.7)	6 (5.8)	1 (5.9)
Allogeneic SCT	8 (3.6)	0	7 (6.8)	1 (5.9)
A(B)VD	13 (5.9)	0	0	13 (76.5)
Asparaginase^a^	13 (5.9)	1 (1.0)	12 (11.7)	0
Splenectomy	1 (0.5)	1 (1.0)	0	0
More than one therapy	41 (18.5)	17 (16.7)	20 (19.4)	4 (23.5)

*B-NHL* B-cell non-Hodgkin lymphoma, *T-NHL* T-cell non-Hodgkin lymphoma, *HL* Hodgkin’s lymphoma, *CS* corticosteroids, *CHO(E)P* combination of cyclophosphamide, hydroxydaunorubicin, vincristine, prednisolone with or without etoposide, *A(B)VD* combination of adriamycin, vinblastine, dacarbazine with or without bleomycin.

Information on HLH-directed treatment and lymphoma-specific therapy was available for 184 and 280 patients, respectively. Results are presented as number of patients and corresponding percentages in parentheses.

^a^In combination.

Treatment of the triggering disease (i.e., lymphoma-directed therapy or combined treatment directed against HLH and lymphoma) resulted in lower mortality (57.7%; vs. 100% mortality for patients who had received HLH-directed therapy only or no treatment at all). Without lymphoma-specific treatment, median estimated survival time was less than 1 month (Supplementary Table 4).

### Prognostic factors, outcome

**Fig. 4:**
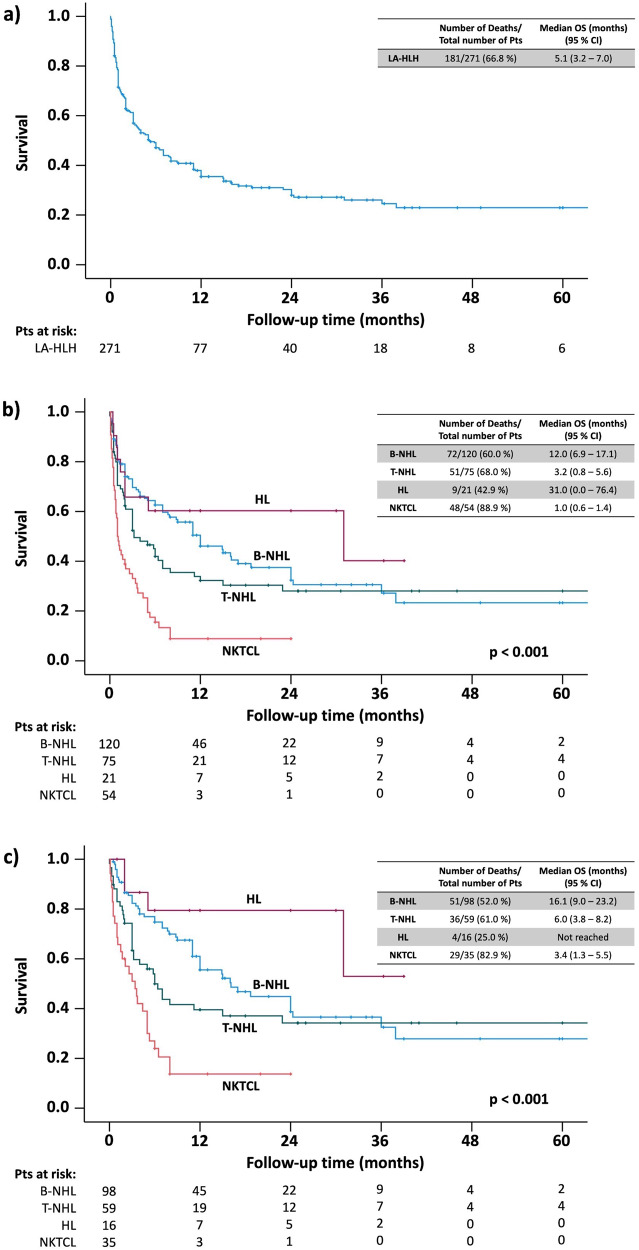
Kaplan-Meier plots displaying estimated survival. Kaplan-Meier plots displaying estimated survival. Plot (**a**) shows information for the entire cohort, (**b**) for lymphoma subgroups, and (**c**) for patients who had received any lymphoma-specific treatment depending on lymphoma subgroup. Abbreviations: LA-HLH lymphoma-associated hemophagocytic lymphohistiocytosis, B-NHL B-cell non-Hodgkin lymphoma, T-NHL T-cell non-Hodgkin lymphoma, HL Hodgkin’s lymphoma, NKTCL natural killer/T-cell lymphoma.

Information on outcome and corresponding follow-up times were available for 271 patients, of whom 181 (66.8%) died after a median estimated survival time of 5.1 months (Fig. 4a). 75 of 271 patients (27.7%) died within the first month after HLH diagnosis, while 48 of these 75 patients (64%) had T-NHL (*N* = 25 NK/T-cell lymphoma, *N* = 23 other T-NHL). Comparing lymphoma subgroups, patients with NK/T-cell lymphoma-HLH had shorter estimated survival time than patients with B-NHL, T-NHL, and Hodgkin’s lymphoma-HLH (1.0 months vs. 12.0, 3.2, and 31.0 months, respectively; *p* < 0.001) (Fig. 4b). Of 222 patients who received lymphoma-specific therapy, 208 had available follow-up data. Median estimated survival time in these patients was 11.0 months, with a particular poor prognosis for patients with T-NHL and NK/T-cell lymphoma (median survival 6.0 and 3.4 months, Fig. 4c). Even though HSCT was carried out only in minority of patients, those receiving HSCT showed considerably longer estimated survival (B-NHL: 36.0 vs. 12.0 months, *p* = 0.004; T-NHL: 93.0 vs. 3.2 months, *p* < 0.001, respectively; Supplementary Fig. 2a, b).

**Fig. 5:**
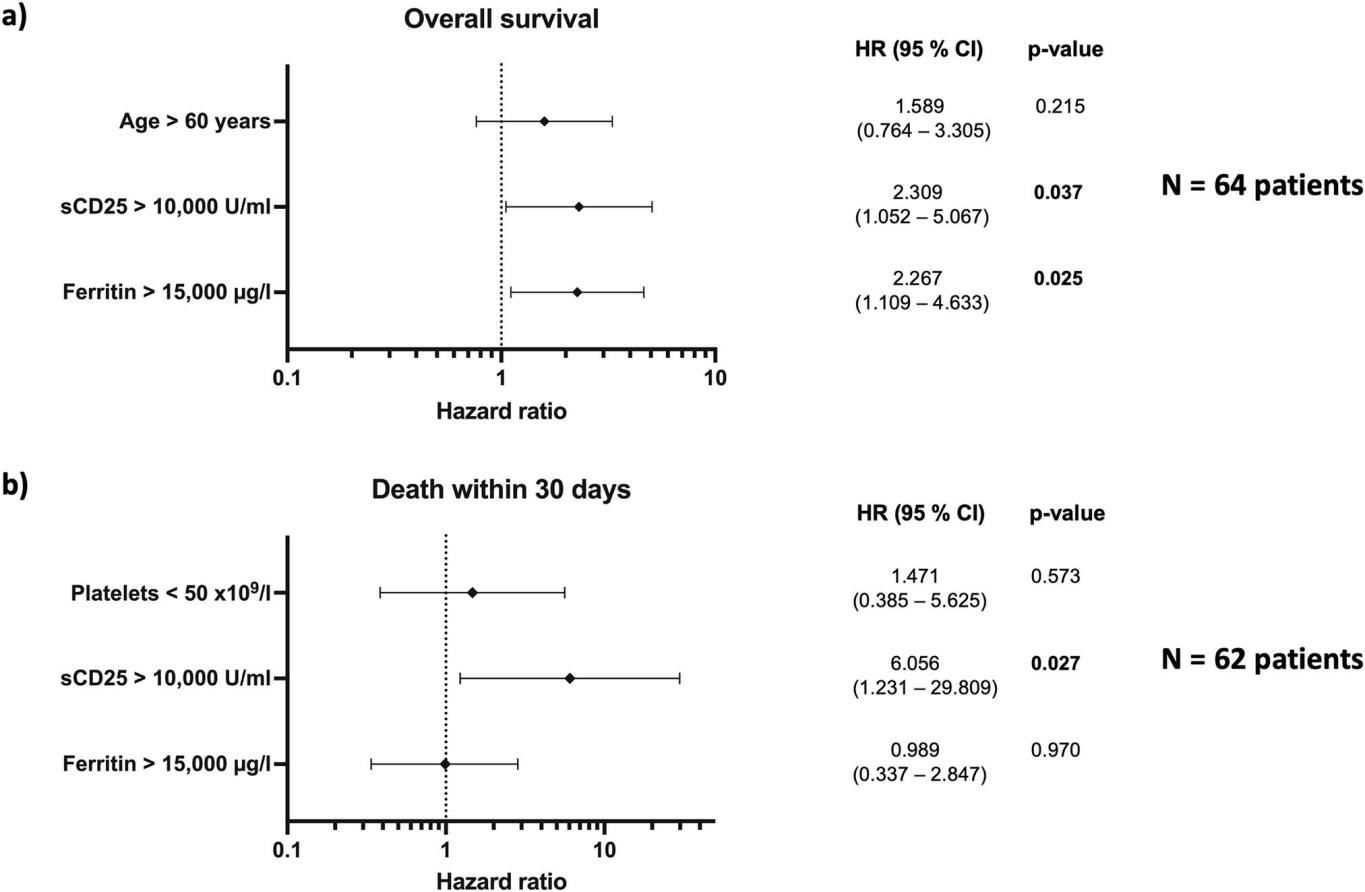
Multivariate Cox regression analysis for possible prognostic factors. Multivariate Cox regression analysis for possible prognostic factors. Results are presented as Forest plots with Hazard ratios and confidence intervals, plots provide information on (**a**) overall mortality and (**b**) 30-day mortality (death within 30 days). Abbreviations: HR hazard ratio, CI confidence interval, sCD25 soluble CD25.

For patients with sufficient data, univariate Cox regression analyses for both overall and 30-day mortality were conducted. Results are summarized in Supplementary Table 5. Age over 60 years, sCD25 levels exceeding 10,000 U/ml, and ferritin levels above 15,000 µg/l were associated with poorer overall survival (OS) in univariate analysis; while sCD25 levels above 10,000 U/ml and platelet counts below 50 × 10^9^/l were associated with poorer 30-day survival. Parameters, which showed statistical significance in univariate analysis were included in a multivariate Cox regression analysis. For 30-day mortality, we also included ferritin above 15,000 µg/l, which shows a tendency towards poorer survival in univariate analysis and is an essential parameter in HLH. The results of the multivariate Cox regression analysis are presented in Fig. 5 as Forest plots displaying hazard ratios and corresponding confidence intervals. Both ferritin levels above 15,000 µg/l and sCD25 levels above 10,000 U/ml independently predicted poorer OS, while the latter one also predicted poorer 30-day survival.

## Discussion

Adult hemophagocytic lymphohistiocytosis (aHLH) is a clinical syndrome characterized by pathologic hyperinflammation caused by aberrant immune activation resulting in a cytokine storm with often fatal organ failure [145, 146]. In most patients, triggering diseases such as infections or malignancies can be identified. With increasing patient age, HLH associated with lymphoma (LA-HLH) is the leading HLH-subtype, typically exhibiting a poorer prognosis compared to HLH following infections or autoimmune diseases [127]. Despite a growing number of published cases or case series during the past years (Supplementary Fig. 1), systematic data on LA-HLH are limited. Within the framework of a scoping review, and to the best of our knowledge, we here present the largest systematic analysis on LA-HLH, comprising data of 132 articles reporting on a total of 542 patients.

The increasing frequency of publications on adult HLH might be due to several reasons. On one hand, we assume higher awareness and diagnostic vigilance, which might be driven mainly by efforts towards a more standardized work-up in HLH during past years (resulting in several published national and international guidelines [9, 147, 148]). On the other hand, there are hints for a rising incidence rate, as reported by a German study [149]. The authors outline a growing number of HLH cases between 2014 and 2020, mainly due to an increase in older HLH patients, for whom there is a higher prevalence of HLH triggers like malignancies and autoimmune diseases. Beyond, this study reports increasing numbers of inpatient deaths related to HLH, possibly attributed to higher age, disease severity and higher lymphoma incidence, harboring especially poor prognosis.

In line with latest epidemiological data on malignant lymphoma and a recent nationwide Swedish study reporting on malignancy-associated HLH, we found a male predominance with 62.7% of all reported LA-HLH cases [150, 151]. Median age at diagnosis in the present cohort was 60 years, while patients with T-NHL-associated HLH were significantly younger compared to those with B-NHL-HLH (49 vs. 64 years, *p* < 0.001). This difference might reflect the significant proportion of NK/T-cell lymphoma within the T-NHL group – a lymphoma subtype with an often particularly young age of onset [152]. Besides NK/T-cell lymphoma, other common subtypes comprised angioimmunoblastic T-cell lymphoma, subcutaneous panniculitis-like T-NHL (SPTCL) and intravascular large B-cell lymphoma (displayed and summarized also in Fig. 3 and Supplementary Table 2). Some of these rare lymphoma subtypes seem to be enriched in LA-HLH patients. Mechanistically, this might be explained by aberrant immune activity of the tumor cells and the tumor microenvironment causing hyperinflammation with cytokine storm, the association of lymphoma pathophysiology with EBV tropism, and/or modulating genetic factors leading to increased risk for HLH development [153, 154]. An example is loss-of-function mutations involving the Hepatitis A Virus-Cellular Receptor 2 gene (*HAVCR2*), which are enriched in SPTCL and result in dysregulated interferon-γ-inducible inflammatory host response as predisposing factor for HLH [155–157]. Of note, germline HAVCR2 mutations have been reported in idiopathic HLH/HLH-like systemic disease, supporting molecular testing in addition to deep skin biopsies in these patients [158, 159]. The discovery of HAVCR2 mutations in inflammatory conditions is a prime example how better understanding of the molecular basis of HLH can lead to treatment optimization, as shown by promising responses following immunomodulatory treatment with the JAK1/2 inhibitor ruxolitinib in SPTCL and patients with lupus panniculitis [159, 160].

Besides more or less disease-specific factors, preexisting iatrogenic or acquired immunosuppression (i.e., infections with human immunodeficiency virus, drugs like steroids or azathioprine) is frequently observed in HLH patients. Consequently, adult HLH may exhibit with variable and occasionally atypical clinical presentation, reflecting the complex pathophysiology and interaction of the aforementioned factors. On the other hand, the multifaceted clinical picture not infrequently leads to delayed or misdiagnosis of HLH, especially if lymphoma-associated.

In this analysis, typical symptoms comprised fever and organomegaly, and nearly all patients with LA-HLH were diagnosed in Ann Arbor stages III and IV. Interestingly, more than half of the patients had lymphoma infiltration in the bone marrow. Moreover, lymphoma infiltration in liver, spleen, and skin was frequently reported. Besides typically aggressive courses and advanced disease stages of lymphoma presenting with HLH, possible explanations might be higher rates of extranodal manifestations in certain lymphoma subtypes enriched in our cohort or preceding immunosuppression, which was shown to significantly increase the risk for aggressive lymphoma (and likewise extranodal manifestations) [161–164]. Even though the presented data have to be interpreted with caution given potential publication bias with a tendency to publish illustrative cases (i.e., with bone marrow infiltration and/or liver/spleen involvement) more frequently, several implications on diagnosis and management of LA-HLH may be drawn. Keeping in mind the especially poor prognosis as demonstrated in this cohort and published studies on LA-HLH [126, 165–167], and even worse survival of less than 1 month if no lymphoma-specific therapy or only HLH-directed therapy is applied (Supplementary Table 4), a comprehensive and vigorous diagnostic work-up with the aim of timely trigger identification is justified. This includes imaging (preferably using positron-emission tomography guidance [168]) and (repetitive) biopsy of suspicious tissue or lymph node excision, especially in patients with no obvious signs of a triggering disease or suspected but yet not proven lymphoma [9]. A recent study from French intensive care specialists demonstrated a high rate of trigger identification in 95% of the examined patients using an aggressive diagnostic approach including tissue biopsies of lymph node, skin, bone marrow, and liver. Of note, it was possible to apply timely trigger specific therapy in 68% of the patients in the Intensive Care Unit [169].

Laboratory hallmarks of HLH comprise cytopenia and in particular highly elevated ferritin and sCD25 levels [170, 171]. In our analysis with special emphasis on LA-HLH, nearly all patients fulfilled the HLH-2004 criteria for ferritin and sCD25 (ferritin >500 µg/l: 90% of all patients, sCD25 ≥ 2400 U/ml: 98% of all patients). Patients with T-NHL-associated HLH had significantly higher ferritin levels compared to those with B-NHL (7645 vs. 2785 µg/l, *p* = 0.033) and presented with lower median platelet counts and fibrinogen, indicating a more profound inflammatory response. Aberrant immune activity and inflammation is a well-known phenomenon in certain T-NHL subtypes, for example NK/T-cell lymphoma, which represent a major group in this report and often go along with EBV-infection [172]. Besides, we observed a particular high rate of EBV replication in the Hodgkin’s lymphoma group (Supplementary Table 3). A strong association of EBV and Hodgkin’s lymphoma-associated HLH was shown in the past, highlighting the role of EBV in the pathogenesis of HLH in this lymphoma subgroup [173]. Active EBV replication acting as a co-trigger should thus be considered in patients with HLH features and suspected malignancy, and there are case reports showing positive EBV-PCR before lymphoma-diagnosis was established [174].

Soluble CD25 (sCD25; synonym: soluble interleukin-2 receptor alpha chain, sIL-2Rα) is a surrogate parameter of T-cell activation and was shown to be a sensitive test in diagnosing adult HLH as well as for monitoring disease activity [171, 175, 176]. In addition, a high sCD25/ferritin ratio was proposed as a marker for the diagnosis of LA-HLH [140]. In our analysis, we found highly elevated median sCD25 levels of about 10,000 U/ml, with highest values in patients with Hodgkin’s lymphoma-associated HLH. Although elevated sCD25 levels are also observed in inflammation, infection, or solid tumors, the values in hematological malignancies (i.e., lymphoma) are typically higher [177]. In an attempt to simplify (and perhaps accelerate) the diagnosis of HLH in hematologic malignancies, Zoref-Lorenz et al. developed a novel combination of ferritin and sCD25, termed optimized HLH inflammatory index (OHI). Using defined cut-offs for ferritin (>1000 µg/l) and sCD25 (>3900 U/ml), patients with malignancy-associated HLH were best identified [11]. In a Japanese study comparing 57 LA-HLH patients to 53 patients with HLH due to benign diseases, a sCD25 cut-off of 5000 U/ml showed best sensitivity and specificity for a diagnosis of LA-HLH [178]. In synopsis of the published literature and the data of our analysis, significantly elevated sCD25 levels in patients with clinical signs of hyperinflammation should prompt diagnostics for a yet undetected lymphoma. Besides the importance in recognizing and diagnosing HLH, sCD25 was recently shown to have also prognostic value in adult HLH [179]. In the present analysis, we demonstrated an adverse prognostic impact for sCD25 levels beyond 10,000 U/ml both for OS and 30-day survival, which might be explained by advanced lymphoma disease and pronounced inflammation leading to organ failure. Given the limited sample size (only 64 and 62 patients eligible for this analysis) and retrospective design of our study, this finding should be interpreted with caution though, and potential consequences (i.e., treatment intensification) should be discussed thoroughly on an individual case basis considering disease severity and potentially harmful treatment effects.

The particular poor prognosis of LA-HLH (this cohort: median estimated survival 5.1 months overall and 11.0 months if lymphoma-specific therapy was applied) warrants further treatment optimization. In the present study, a detailed analysis for example on the early use of etoposide was not feasible due to limited data availability. However, the dismal prognosis of patients not receiving any lymphoma-specific therapy and a patient proportion of almost 30% dying within the first month after HLH diagnosis indicates the need of combined anti-inflammatory (HLH-directed) and lymphoma-specific therapy as soon as possible. LA-HLH patients often present with severe disease characterized by imminent or manifest organ failure clinically and highly elevated ferritin and sCD25 levels as laboratory surrogate parameters. In these severely ill patients, profound immunosuppression beyond steroids and polyvalent immunoglobulins is typically necessary in order to effectively suppress hyperinflammation, thus allowing for further diagnostics and to bridge the time gap between HLH diagnosis and begin of lymphoma-specific therapy. In this situation, current guidelines recommend adding dose-adjusted etoposide (i.e., 50–100 mg/m^2^ once or twice weekly) [8, 9, 180] given its activity against inflammation and lymphoma. This approach is supported by several studies showing beneficial effects for early use of etoposide in patients with LA-HLH [126, 181]. However, large prospective studies are lacking with respect to rarity of LA-HLH and often dramatic clinical disease courses. Thus, we strongly encourage international collaborations to study aspects of optimal treatment systematically in a larger cohort of well-characterized lymphoma-HLH patients, in particular with the advent of more targeted (and maybe less harmful) therapies like ruxolitinib [182].

Beneficial effects for incorporating etoposide in subsequent lymphoma-therapy have also been suggested, a recent Chinese study demonstrated high overall response rates and promising survival data in patients with B-NHL-HLH (5-year overall survival 73%) treated with dose-adjusted EPOCH-R regimen in first-line [183]. This observation is in line with previous reports on high-risk patients with aggressive B-NHL, showing 4-year OS of 75% [184] and 10-year OS of 72% for patients treated with R-CHOEP-14 [185]. Based on these data, inclusion of etoposide in first line therapy seems reasonable in LA-HLH patients, especially in younger patients with B-NHL triggered HLH.

As demonstrated within this cohort, lymphoma presenting with HLH typically exhibit aggressive disease courses and poor prognosis even if initial cytokine storm is controlled and lymphoma-specific therapy is applied (median survival 11 months for these patients in the present analysis). For that reason, primary consolidation therapy using high-dose chemotherapy and subsequent stem cell transplantation is proposed in guidelines, even though these considerations are mainly derived from a study on peripheral T-NHL [186]. Data regarding this special issue are very limited and primarily case reports. In the present analysis, 29 patients had HSCT (SCT; *N* = 21 autologous, *N* = 8 allogeneic) and exhibited a considerably better prognosis. Of note, all patients in the B-NHL group (*N* = 14) underwent autologous SCT. In line with these findings, a recent study by Song et al. reported encouraging OS and similar prognosis for LA-HLH patients undergoing autologous SCT when compared to lymphoma patients without HLH undergoing autologous SCT [187]. In this study, all patients received conditioning regimen with cyclophosphamide, BCNU, and etoposide. Obviously, these data are to some extent preliminary and have to be interpreted with caution. However, in the absence of more sophisticated data, consolidating high-dose chemotherapy and autologous SCT (or initiation of donor search for allogeneic SCT in patients with disseminated NK/T-cell lymphoma) should be evaluated depending on the individual patient’s constitution in B-NHL- and T-NHL-HLH.

Our study has several limitations: first of all, retrospective design and limited data availability depending on published information prevented several more detailed analyses. Moreover, a publication bias with a tendency to publish notably illustrative cases (as discussed above) cannot be ruled out. Despite these restrictions, we here to the best of our knowledge present the largest analysis on LA-HLH, providing a comprehensive overview especially regarding to lymphoma subtypes, clinical presentation, and laboratory features. The especially poor prognosis in LA-HLH patients was confirmed even if lymphoma-specific therapy is applied, justifying an aggressive diagnostic approach and underlining the need of larger and prospective studies to determine and refine optimal management in lymphoma-associated HLH.

### Supplementary information

Supplementary Material
