# Supplementary material for: Lymphoma-associated hemophagocytic lymphohistiocytosis (LA-HLH): a scoping review unveils clinical and diagnostic patterns of a lymphoma subgroup with poor prognosis
Source: Leukemia. 2024 Jan 18;38(2):235–49. doi: 10.1038/s41375-024-02135-8 (PMC10844097; doi:10.1038/s41375-024-02135-8)
Supplement: Supplementary file 1 — Supplementary Material [file 41375_2024_2135_MOESM1_ESM.pdf]

Supplementary information

Lymphoma-associated hemophagocytic lymphohistiocytosis (LA-HLH): a scoping review unveils clinical and diagnostic patterns of a lymphoma subgroup with poor prognosis

Johanna Knauft[1], Thomas Schenk[1], Thomas Ernst[1], Ulf Schnetzke[1], Andreas Hochhaus[1], Paul La Rosée[2], Sebastian Birndt[1]

[1] Klinik für Innere Medizin II, Hämatologie und internistische Onkologie, Universitätsklinikum Jena, Jena, Germany
[2] Klinik für Innere Medizin II, Onkologie, Hämatologie, Immunologie, Infektiologie und Palliativmedizin, Schwarzwald-Baar Klinikum, Villingen-Schwenningen, Germany

Table of contents

Supplemental Figure legends 2
Supplemental Figure 1 3
Supplemental Figure 2 4
Supplemental Table 1 5
Supplemental Table 2 6
Supplemental Table 3 7
Supplemental Table 4 7
Supplemental Table 5 8
Supplemental Table 6 9
Appendix 1 – List of references included in detailed analysis 11

Supplemental Figure legends

Supplemental Figure 1

Number of initially gathered articles (N = 595) with corresponding years of publication. Articles included for further analysis in the present study are highlighted in blue (N = 132).

Supplemental Figure 2

Kaplan-Meier plots showing estimated survival for a) patients with B-NHL and b) patients with T-NHL who underwent hematopoietic stem cell transplantation (SCT) versus those who did not. A p-value below 0.05 was considered statistically significant. Information on patients at risk for different time points is provided below the curves.

Supplemental Figure 1

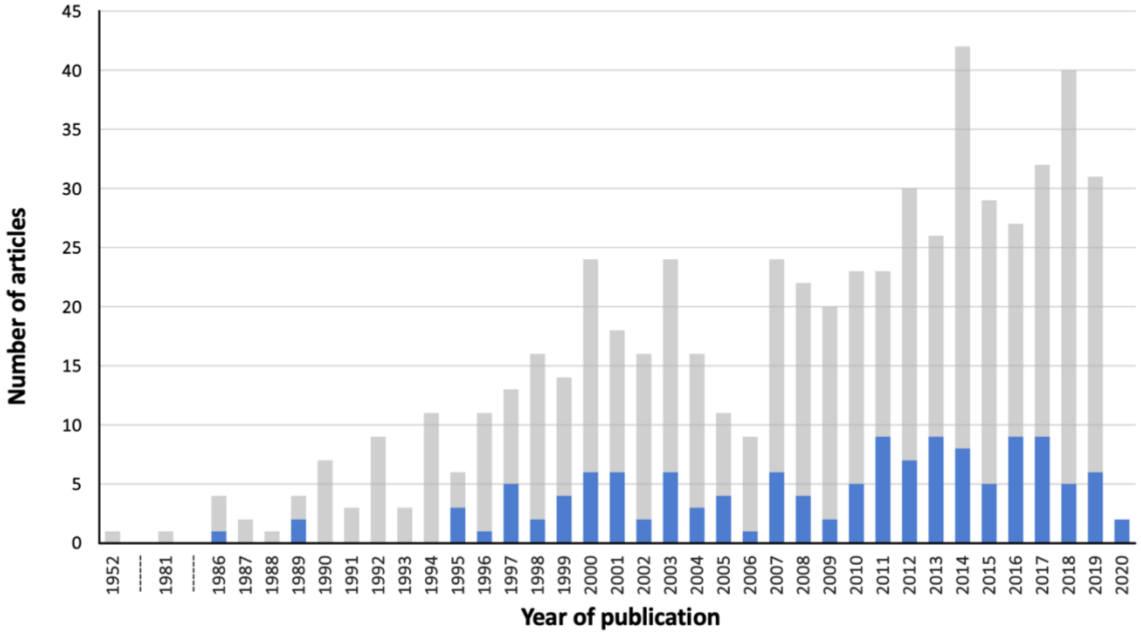

Supplemental Figure 2

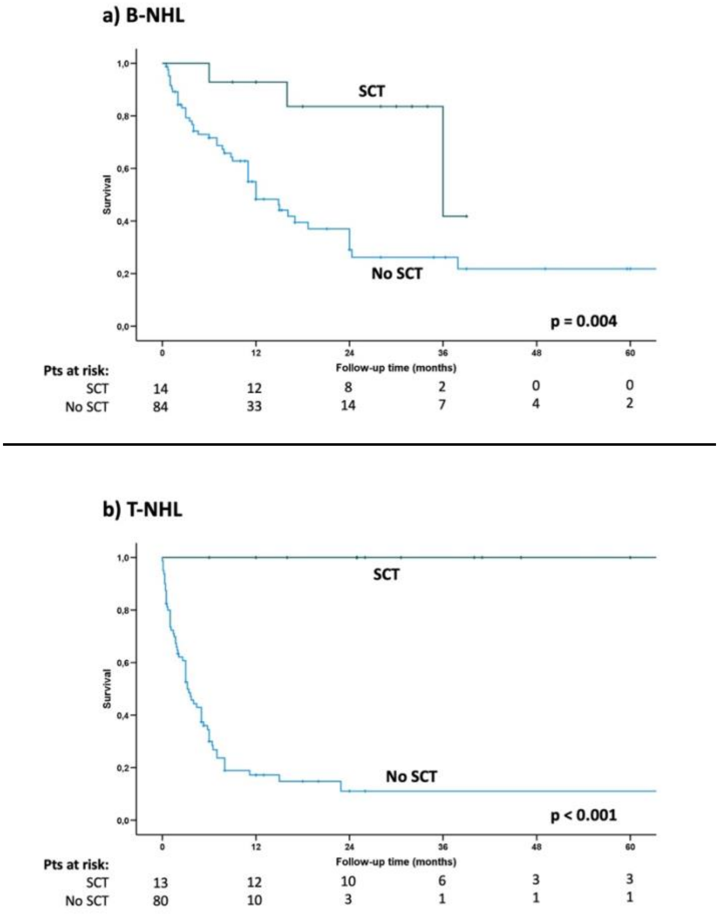

Supplemental Table 1

Reported lymphoma-associated HLH cases according to country of origin and corresponding lymphoma subtype.

	**T-NHL**	PTCL	AITL	ALCL	NKTCL	Other TCL	**B-NHL**	DLBCL	MZL	MCL	LPL	HGBCL	CLL	BCL NOS	**HL**	**Unspecified**	
Japan	**52**	19	4	4	22	3	**116**	96	2	0	0	0	0	18	**6**	**0**	**174**
China	**106**	38	7	0	61	0	**32**	26	0	1	0	1	2	2	**0**	**0**	**138**
France	**27**	8	1	3	4	11	**47**	22	1	0	2	0	0	22	**18**	**1**	**93**
Germany	**10**	3	2	1	3	1	**21**	12	2	1	0	0	2	4	**7**	**1**	**39**
Mexico	**18**	9	1	2	6	0	**9**	7	0	0	0	0	0	2	**6**	**0**	**33**
USA	**10**	4	0	1	4	1	**11**	9	0	0	0	1	0	1	**4**	**0**	**25**
United Kingdom	**2**	1	0	1	0	0	**3**	2	0	0	0	0	1	0	**1**	**0**	**6**
South Korea	**2**	0	0	1	1	0	**3**	3	0	0	0	0	0	0	**0**	**0**	**5**
Thailand	**4**	4	0	0	0	0	**1**	1	0	0	0	0	0	0	**0**	**0**	**5**
Spain	**3**	0	0	0	2	1	**0**	0	0	0	0	0	0	0	**1**	**0**	**4**
Australia	**1**	0	0	1	0	0	**1**	1	0	0	0	0	0	0	**1**	**0**	**3**
Canada	**2**	1	0	1	0	0	**0**	0	0	0	0	0	0	0	**0**	**0**	**2**
Italy	**1**	1	0	0	0	0	**1**	1	0	0	0	0	0	0	**0**	**0**	**2**
Switzerland	**0**	0	0	0	0	0	**0**	0	0	0	0	0	0	0	**2**	**0**	**2**
Sweden	**1**	0	0	1	0	0	**0**	0	0	0	0	0	0	0	**1**	**0**	**2**
Turkey	**2**	1	0	1	0	0	**0**	0	0	0	0	0	0	0	**0**	**0**	**2**
The Netherlands	**0**	0	0	0	0	0	**0**	0	0	0	0	0	0	0	**1**	**0**	**1**
Belgium	**1**	0	0	0	0	1	**0**	0	0	0	0	0	0	0	**0**	**0**	**1**
Austria	**1**	1	0	0	0	0	**0**	0	0	0	0	0	0	0	**0**	**0**	**1**
Czech Republic	**1**	1	0	0	0	0	**0**	0	0	0	0	0	0	0	**0**	**0**	**1**
Israel	**1**	0	0	1	0	0	**0**	0	0	0	0	0	0	0	**0**	**0**	**1**
Tunisia	**0**	0	0	0	0	0	**1**	1	0	0	0	0	0	0	**0**	**0**	**1**
Malaysia	**0**	0	0	0	0	0	**1**	1	0	0	0	0	0	0	**0**	**0**	**1**
	**245**	91	15	18	103	18	**247**	182	5	2	2	2	5	49	**48**	**2**	**542**

Abbreviations: T-NHL, T-cell non-Hodgkin lymphoma; PTCL, Peripheral T-cell lymphoma; AITL, Angioimmunoblastic T-cell lymphoma; ALCL, Anaplastic large-cell lymphoma; NKTCL, Natural killer/T-cell lymphoma; B-NHL, B-cell non-Hodgkin lymphoma; DLBCL, Diffuse large B-cell lymphoma; MZL, Marginal zone lymphoma; MCL, Mantle cell lymphoma; LPL, Lymphoplasmacytic lymphoma; HGBCL, High-grade B-cell lymphoma; CLL, B-cell Chronic lymphocytic leukemia; BCL NOS, B-cell lymphoma, not otherwise specified; HL, Hodgkin´s lymphoma

Supplemental Table 2

Detailed list and percentage distribution of 542 lymphoma cases associated with HLH according to 2016 World Health Organization classification of lymphoid neoplasms.

	N (%)
B-cell non-Hodgkin lymphoma	***247 (45.6)***
Diffuse large B-cell lymphoma, not otherwise specified	119 (48.2)
T cell/histiocyte-rich large B-cell lymphoma	4 (1.6)
Primary diffuse large B-cell lymphoma of the central nervous system	1 (0.4)
EBV+ diffuse large B-cell lymphoma, not otherwise specified	1 (0.4)
Intravascular large B-cell lymphoma	57 (23.1)
High-grade B-cell lymphoma with MYC and BCL2 and/or BCL6 rearrangements	1 (0.4)
High-grade B-cell lymphoma, not otherwise specified	1 (0.4)
Lymphoplasmacytic lymphoma	2 (0.8)
Extranodal marginal zone lymphoma of mucosa-associated lymphoid tissue	1 (0.4)
Nodal marginal zone lymphoma	1 (0.4)
Splenic marginal zone lymphoma	1 (0.4)
Marginal zone lymphoma, not otherwise specified	2 (0.8)
Mantle cell lymphoma	2 (0.8)
Chronic lymphocytic leukemia	5 (2.0)
B-cell lymphoma, not otherwise specified	49 (19.8)
T-cell non-Hodgkin lymphoma	***245 (45.2)***
Peripheral T-cell lymphoma, not otherwise specified	52 (21.2)
Subcutaneous panniculitis-like T-cell lymphoma	21 (8.6)
Primary cutaneous gamma/delta T-cell lymphoma	2 (0.8)
Primary cutaneous CD8+ aggressive epidermotropic cytotoxic T-cell lymphoma	1 (0.4)
Sézary syndrome	2 (0.8)
Mycosis fungoides	4 (1.6)
Hepatosplenic T-cell lymphoma	6 (2.4)
Systemic EBV+ T-cell lymphoma of childhood	1 (0.4)
Enteropathy-associated T-cell lymphoma	1 (0.4)
T-cell large granular lymphocytic leukemia	1 (0.4)
Extranodal natural killer/T-cell lymphoma, nasal type	73 (29.8)
Natural killer/T-cell lymphoma, not otherwise specified	30 (12.2)
Angioimmunoblastic T-cell lymphoma	15 (6.1)
Anaplastic large-cell lymphoma, ALK+	9 (3.7)
Anaplastic large-cell lymphoma, ALK-	9 (3.7)
T-cell lymphoblastic leukemia/lymphoma	1 (0.4)
T-cell lymphoma, not otherwise specified	17 (6.9)
Hodgkin´s lymphoma	***48 (8.9)***
Classical Hodgkin lymphoma	17 (34.7)
Mixed cellularity classical Hodgkin lymphoma	4 (23.5)
Nodular sclerosis classical Hodgkin lymphoma	3 (17.6)
Lymphocyte-depleted classical Hodgkin lymphoma	2 (11.8)
Lymphocyte-rich classical Hodgkin lymphoma	1 (5.9)
Classical Hodgkin lymphoma, not otherwise specified	7 (41.2)
Hodgkin lymphoma without further information on subtype	31 (64.6)
Unspecified lymphoma	***2 (0.4)***

Abbreviations: EBV, Epstein-Barr virus; CD, cluster of differentiation; ALK, anaplastic lymphoma kinase

Supplemental Table 3

Epstein-Barr virus status of Lymphoma-HLH patients. Data are provided as number of patients and corresponding percentages.

	Total	B-NHL	T-NHL	HL
**Number of patients**	131	64	48	19
**EBV negative**	53 (40.5)	31 (48.4)	20 (41.7)	2 (10.5)
**Primary (recent) infection**	0 (0)	0 (0)	0 (0)	0 (0)
**Past infection**	29 (22.1)	21 (32.8)	8 (16.7)	0 (0.0)
**Reactivated**	49 (37.4)	12 (18.8)	20 (41.7)	17 (89.5)

Abbreviations: EBV, Epstein-Barr virus; B-NHL, B-cell non-Hodgkin lymphoma; T-NHL, T-cell non-Hodgkin lymphoma; HL, Hodgkin´s lymphoma

Supplemental Table 4

Impact of lymphoma-specific treatment in LA-HLH patients. Information on outcome, follow-up time and lymphoma treatment were available for 264 patients, of whom 176 (66.7 %) died.

	Total N = 264 pts	Death n/N (%)	Median estimated survival time in months (95 % CI)
**No treatment**	16 (6.1 %)	16/16 (100.0)	0.5 (0.11 – 0.89)
**Treatment**	248 (93.9 %)	160/248 (64.5)	6.5 (4.6 – 8.4)
**Only HLH-directed**	40 (15.2 %)	40/40 (100.0)	0.8 (0.56 – 1.05)
**No lymphoma specific therapy**	56 (21.2 %)	56/56 (100.0)	0.7 (0.53 – 0.88)
**Lymphoma specific therapy with or without HLH therapy**	208 (78.8 %)	120/208 (57.7)	11.0 (6.2 – 15.8)

Abbreviations: HLH, hemophagocytic lymphohistiocytosis; pts, patients; CI, Confidence interval

Supplemental Table 5

Univariate Cox regression analysis for a) overall and b) 30-day mortality. Results are presented as Hazard ratio with corresponding confidence intervals.

a) Overall mortality			
	**Hazard ratio**	**95 % Confidence interval**	**p-value**
Age > 60 years	1.534	1.016 – 2.316	0.042*+
Sex (female vs. male)	1.168	0.774 – 1.761	0.459
sCD25 > 10,000 U/ml	3.004	1.422 – 6.348	0.004*+
Ferritin > 5,000 µg/l	1.523	0.913 – 2.540	0.107
Ferritin > 15,000 µg/l	2.364	1.447 – 3.861	<0.001*+
sCD25/Ferritin ratio > 2.0	0.957	0.469 – 1.952	0.903
Hemoglobin < 90 g/l	0.889	0.566 – 1.397	0.609
ANC < 1 x 10^9/l	1.746	0.853 – 3.572	0.127
Platelet count < 50 x 10^9/l	1.279	0.843 – 1.938	0.247
Platelet count < 20 x 10^9/l	1.330	0.773 – 2.286	0.303
Cytopenia ≥ 2 lineages	0.918	0.590 – 1.429	0.704
Bilirubin > 1.1 mg/dl	1.736	0.812 – 3.713	0.155
Fibrinogen < 1.5 g/l	1.262	0.678 – 2.349	0.463
Triglycerides ≥ 265 mg/dl	1.870	0.814 – 4.296	0.140

b) 30-day mortality			
	**Hazard ratio**	**95 % Confidence interval**	**p-value**
Age > 60 years	1.514	0.849 – 2.701	0.160
Sex (female vs. male)	1.051	0.589 – 1.874	0.866
sCD25 > 10,000 U/ml	6.668	1.491 – 29.820	0.013*+
Ferritin > 5,000 µg/l	1.501	0.723 – 3.113	0.276
Ferritin > 15,000 µg/l	1.944	0.972 – 3.888	0.060+
sCD25/Ferritin ratio > 2.0	1.746	0.612 – 4.982	0.297
Hemoglobin < 90 g/l	0.745	0.404 – 1.373	0.345
ANC < 1 x 10^9/l	1.489	0.554 – 4.001	0.430
Platelet count < 50 x 10^9/l	2.301	1.233 – 4.292	0.009*+
Platelet count < 20 x 10^9/l	1.508	0.725 – 3.137	0.272
Cytopenia ≥ 2 lineages	0.844	0.456 – 1.563	0.589
Bilirubin > 1.1 mg/dl	7.312	0.969 – 55.178	0.054
Fibrinogen < 1.5 g/l	1.283	0.554 – 2.972	0.560
Triglycerides ≥ 265 mg/dl	1.284	0.422 – 3.900	0.660

Legends:
* indicates statistically significant values
+ indicates variables used in multivariate Cox regression analysis

Abbreviations: sCD25, Soluble CD25; ANC, Absolute neutrophil count; LDH, Lactate dehydrogenase

Supplemental Table 6
Laboratory characteristics of patients with Hodgkin´s lymphoma-associated HLH.

		N or n/N (%) or Median (Range)
Number of patients		24
Male		18/24 (75.0)
Age (years)	n = 24	60 [29-79]
Anemia (Hemoglobin <90 g/l)		21/24 (87.5)
Hemoglobin (g/l)	n = 24	76 [52-110]
Leukopenia (WBC count $<4x10^9/l$)		15/17 (88.2)
Leukocyte count ($x10^9/l$)	n = 17	1.6 [0.3-9.0]
Neutropenia (ANC $<1x10^9/l$)		6/10 (60.0)
ANC $<0.5x10^9/l$		2/8 (25.0)
Neutrophils ($x10^9/l$)	n = 8	1.0 [0.1-3.6]
Thrombocytopenia (Platelets $<100x10^9/l$)		23/24 (95.8)
Platelets $<20x10^9/l$		5/24 (20.8)
Platelet count ($x10^9/l$)	n = 24	36 [4-133]
Cytopenia (≥2 lineages)		21/24 (87.5)
Hyperferritinemia (≥500 µg/l)		24/24 (100.0)
Ferritin >1000 µg/l		24/24 (100.0)
Ferritin >10,000 µg/l		14/24 (58.3)
Ferritin >15,000 µg/l		11/24 (45.8)
Ferritin (µg/l)	n = 24	13,121 [1,100-102,893]
Hypertriglyceridemia (≥265 mg/dl)		8/16 (50.0)
Triglycerides (mg/dl)	n = 16	281 [75-742]
Hypofibrinogenemia (<150 mg/dl)		2/13 (15.4)
Fibrinogen (mg/dl)	n = 13	360 [0-561]
Soluble CD25 (≥2,400 U/ml)		12/12 (100.0)
sCD25 >3,900 U/ml		12/12 (100.0)
sCD25 >10,000 U/ml		8/12 (66.7)
sCD25 (U/ml)	n = 12	24,414 [4,250-96,415]
sCD25/ferritin ratio	n = 12	1.30 [0.24-19.18]
ALAT > ULN		15/18 (83.3)
ALAT > 5x ULN		4/15 (26.7)
ALAT > 10x ULN		0/15 (0)
ASAT > ULN		13/16 (81.3)
ASAT > 5x ULN		4/15 (26.7)
ASAT > 10x ULN		0/15 (0)
Hypoalbuminemia (<35 g/l)		10/10 (100.0)
Albumin (g/l)	n = 10	17 [11-26]
Total bilirubin >1.1 mg/dl		7/8 (87.5)
Total bilirubin (mg/dl)	n = 8	14.0 [0.7-40.6]
LDH > ULN		16/17 (94.1)
LDH > 5x ULN		2/16 (12.5)
LDH > 10x ULN		0/16 (0)

Abbreviations: WBC, white blood cell count*;* ANC, absolute neutrophil count; sCD25, Soluble CD25; ALAT, alanine aminotransferase; ASAT, aspartate aminotransferase; LDH, Lactate dehydrogenase; ULN, upper limit of normal

Appendix 1.

List of references included in detailed analysis [1-132] (N = 132, alphabetical order)

1. Adler NR, Sia CS, Polchleb C, Jane S, Aung AK. Intravascular large B cell lymphoma with haemophagocytic syndrome: a double lethal masquerade. *Intern Med J* 2015 Dec; **45**(12)**:** 1310-1312.

2. Alaoua A, Gilbert G, Ghannouchi N, Houchlef M, Letaief A, Bahri F. Primary bilateral adrenal lymphoma revealed by hemophagocytic syndrome. *Ann Endocrinol (Paris)* 2011 Jun; **72**(3)**:** 247-250.

3. Aljitawi OS, Boone JM. Lymphoma-associated hemophagocytic lymphohistiocytosis. *Blood* 2012 Aug 2; **120**(5)**:** 932.

4. Anghel G, Petrinato G, Severino A, Remotti D, Insabato L, De Renzo A*, et al.* Intravascular B-cell lymphoma: report of two cases with different clinical presentation but rapid central nervous system involvement. *Leuk Lymphoma* 2003 Aug; **44**(8)**:** 1353-1359.

5. Apodaca E, Rodriguez-Rodriguez S, Tuna-Aguilar EJ, Demichelis-Gomez R. Prognostic Factors and Outcomes in Adults With Secondary Hemophagocytic Lymphohistiocytosis: A Single-center Experience. *Clin Lymphoma Myeloma Leuk* 2018 Oct; **18**(10)**:** e373-e380.

6. Bailey C, Dearden C, Ardeshna K. Haemophagocytic lymphohistiocytosis as a consequence of untreated B-cell chronic lymphocytic leukaemia. *BMJ Case Rep* 2017 Apr 26; **2017**.

7. Bains A, Mamone L, Aneja A, Bromberg M. Lymphoid malignancy-associated hemophagocytic lymphohistiocytosis: Search for the hidden source. *Annals of diagnostic pathology* 2017 Jun; **28:** 37-42.

8. Baselga E, Pujol RM, Costa I, Bordas R, De Moragas JM. Subcutaneous angiocentric T-cell lymphoma associated with fatal hemophagocytic syndrome. *Int J Dermatol* 1997 May; **36**(5)**:** 363-367.

9. Bhagwati NS, Oiseth SJ, Abebe LS, Wiernik PH. Intravascular lymphoma associated with hemophagocytic syndrome: a rare but aggressive clinical entity. *Ann Hematol* 2004 Apr; **83**(4)**:** 247-250.

10. Bigenwald C, Fardet L, Coppo P, Meignin V, Lazure T, Fabiani B*, et al.* A comprehensive analysis of Lymphoma-associated haemophagocytic syndrome in a large French multicentre cohort detects some clues to improve prognosis. *Br J Haematol* 2018 Oct; **183**(1)**:** 68-75.

11. Birndt S, Schenk T, Heinevetter B, Brunkhorst FM, Maschmeyer G, Rothmann F*, et al.* Hemophagocytic lymphohistiocytosis in adults: collaborative analysis of 137 cases of a nationwide German registry. *J Cancer Res Clin Oncol* 2020 Apr; **146**(4)**:** 1065-1077.

12. Blom A, Beylot-Barry M, D'Incan M, Laroche L. Lymphoma-associated hemophagocytic syndrome (LAHS) in advanced-stage mycosis fungoides/Sezary syndrome cutaneous T-cell lymphoma. *J Am Acad Dermatol* 2011 Aug; **65**(2)**:** 404-410.

13. Boland PJ, Hegerova LT, Williams SJ, McKenna RW, Bachanova V, Eckfeldt CE. Successful treatment of two cases of classical Hodgkin lymphoma-associated hemophagocytic lymphohistiocyosis with R-CEPP. *Leuk Lymphoma* 2017 Feb; **58**(2)**:** 478-481.

14. Brown NA, Ross CW, Gudjonsson JE, Wale D, Pawarode A, Maillard I*, et al.* Subcutaneous panniculitis-like T-cell lymphoma with bone marrow involvement. *Am J Clin Pathol* 2015 Feb; **143**(2)**:** 265-273.

15. Chan EY, Pi D, Chan GT, Todd D, Ho FC. Peripheral T-cell lymphoma presenting as hemophagocytic syndrome. *Hematol Oncol* 1989 Jul-Aug; **7**(4)**:** 275-285.

16. Cheng FY, Tsui WM, Yeung WT, Ip LS, Ng CS. Intravascular lymphomatosis: a case presenting with encephalomyelitis and reactive haemophagocytic syndrome diagnosed by renal biopsy. *Histopathology* 1997 Dec; **31**(6)**:** 552-554.

17. Chhabra S, Strair RK, Rubin AD. Haemophagocytic lymphohistiocytosis and primary central nervous system lymphoma. *Intern Med J* 2013 Apr; **43**(4)**:** 463-464.

18. Cho SG, Koh YB, Chang HS, Park G, Kang CS, Park JW*, et al.* Successful treatment with splenectomy and interferon alpha against recurred hemophagocytic syndrome in remission state of anaplastic large cell lymphoma following high-dose therapy and autologous peripheral blood stem cell transplantation. *Eur J Haematol* 2005 Mar; **74**(3)**:** 259-262.

19. Chubachi A, Miura I, Hatano Y, Ohshima A, Nishinari T, Miura AB. Syndrome of inappropriate secretion of antidiuretic hormone in patients with lymphoma-associated hemophagocytic syndrome. *Ann Hematol* 1995 Jan; **70**(1)**:** 53-55.

20. Ciaudo M, Chauvenet L, Audouin J, Rossert J, Favier R, Horellou MH*, et al.* Peripheral-T-cell lymphoma with hemophagocytic histiocytosis localised to the bone marrow associated with inappropriate secretion of antidiuretic hormone. *Leuk Lymphoma* 1995 Nov; **19**(5-6)**:** 511-514.

21. Cuttelod M, Pascual A, Baur Chaubert AS, Cometta A, Osih R, Duchosal MA*, et al.* Hemophagocytic syndrome after highly active antiretroviral therapy initiation: a life-threatening event related to immune restoration inflammatory syndrome? *Aids* 2008 Feb 19; **22**(4)**:** 549-551.

22. Davidson-Moncada JK, McDuffee E, Roschewski M. CD5+ diffuse large B-cell lymphoma with hemophagocytosis. *J Clin Oncol* 2013 Feb 20; **31**(6)**:** e76-79.

23. Dawson L, den Ottolander GJ, Kluin PM, Leeksma O. Reactive hemophagocytic syndrome as a presenting feature of Hodgkin's disease. *Ann Hematol* 2000 Jun; **79**(6)**:** 322-326.

24. Demirkan F, Vural F, Ozsan GH, Ozcan MA, Ozkal S, Undar B. Hemophagocytic syndrome associated with inappropiate secretion of antidiuretic hormone in lymphoma and acute myeloblastic leukemia: report of two cases. *Leuk Lymphoma* 2001 Nov-Dec; **42**(6)**:** 1401-1404.

25. Dominguez-Munoz MA, Morales-Camacho RM, Prats-Martin C, Avila R, Vargas MT, Burillo S*, et al.* Unusual co-occurrence of Hodgkin lymphoma and hemophagocytic lymphohistiocytosis in a bone marrow aspirate. *Ann Hematol* 2016 May; **95**(6)**:** 1019-1021.

26. Dufau JP, Le Tourneau A, Molina T, Le Houcq M, Claessens YE, Rio B*, et al.* Intravascular large B-cell lymphoma with bone marrow involvement at presentation and haemophagocytic syndrome: two Western cases in favour of a specific variant. *Histopathology* 2000 Dec; **37**(6)**:** 509-512.

27. Epperla N, Harrington AM, Hemauer K, Shah NN. Extracavitary primary effusion lymphoma associated with hemophagocytic lymphohistiocytosis. *Am J Hematol* 2016 Nov; **91**(11)**:** 1161-1164.

28. Eser B, Altuntas F, Er O, Kontas O, Ferahbas A, Cetin M*, et al.* A case of subcutaneous panniculitis-like T-cell lymphoma with haemophagocytosis developing secondary to chemotherapy. *J Eur Acad Dermatol Venereol* 2004 Nov; **18**(6)**:** 713-715.

29. Flew SJ, Radcliffe KW. Haemophagocytic lymphohistiocytosis complicating Hodgkin's lymphoma in an HIV-positive individual. *Int J STD AIDS* 2010 Aug; **21**(8)**:** 601-603.

30. Fretwell TB, Hanna M. An incidental finding of severe hyperferritinaemia: a lesson to be learned. *J R Coll Physicians Edinb* 2018 Mar; **48**(1)**:** 30-32.

31. Fung KM, Chakrabarty JH, Kern WF, Magharyous H, Gehrs BC, Li S. Intravascular large B-cell lymphoma with hemophagocytic syndrome (Asian variant) in a Caucasian patient. *Int J Clin Exp Pathol* 2012; **5**(5)**:** 448-454.

32. Gerard L, Oksenhendler E. Hodgkin's lymphoma as a cause of fever of unknown origin in HIV infection. *AIDS Patient Care STDS* 2003 Oct; **17**(10)**:** 495-499.

33. Ghose A, Yellu M, Wise-Draper T, Sharma D, Qualtieri J, Latif T*, et al.* Lymphoma presenting as secondary HLH: a review with a tale of two cases. *Clin Lymphoma Myeloma Leuk* 2014 Dec; **14**(6)**:** e187-193.

34. Greil C, Roether F, La Rosee P, Grimbacher B, Duerschmied D, Warnatz K. Rescue of Cytokine Storm Due to HLH by Hemoadsorption in a CTLA4-Deficient Patient. *J Clin Immunol* 2017 Apr; **37**(3)**:** 273-276.

35. Hagihara M, Inoue M, Hua J, Iwaki Y. Lymphocyte-depleted Hodgkin lymphoma complicating hemophagocytic lymphohistiocytosis as an initial manifestation: a case report and review of the literature. *Intern Med* 2012; **51**(21)**:** 3067-3072.

36. Han SM, Teng CL, Hwang GY, Chou G, Tsai CA. Primary splenic lymphoma associated with hemophagocytic lymphohistiocytosis complicated with splenic rupture. *J Chin Med Assoc* 2008 Apr; **71**(4)**:** 210-213.

37. Hanaoka M, Tsukimori K, Hojo S, Abe Y, Mutou T, Muta K*, et al.* B-cell lymphoma during pregnancy associated with hemophagocytic syndrome and placental involvement. *Clin Lymphoma Myeloma* 2007 Jul; **7**(7)**:** 486-490.

38. Harada S, Shinohara T, Naruse K, Machida H. Diffuse 18F-fluorodeoxyglucose accumulation in the bone marrow of a patient with haemophagocytic lymphohistiocytosis due to Hodgkin lymphoma. *BMJ Case Rep* 2016 Oct 28; **2016**.

39. Harvey Y, Wordsworth H, Sia H. Epstein-Barr virus-negative Hodgkin lymphoma presenting as haemophagocytic lymphohistiocytosis. *Br J Haematol* 2015 Apr; **169**(1)**:** 2.

40. He M, Jia J, Zhang J, Beejadhursing R, Mwamaka Sharifu L, Yu J*, et al.* Pregnancy-associated hemophagocytic lymphohistiocytosis secondary to NK/T cells lymphoma: A case report and literature review. *Medicine (Baltimore)* 2017 Nov; **96**(47)**:** e8628.

41. Hirai H, Shimazaki C, Hatsuse M, Okano A, Ashihara E, Inaba T*, et al.* Autologous peripheral blood stem cell transplantation for adult patients with B-cell lymphoma-associated hemophagocytic syndrome. *Leukemia* 2001 Feb; **15**(2)**:** 311-312.

42. Hrudka J, Eis V, Herman J, Prouzova Z, Rosenwald A, Duska F. Panniculitis-like T-cell-lymphoma in the mesentery associated with hemophagocytic syndrome: autopsy case report. *Diagn Pathol* 2019 Jul 17; **14**(1)**:** 80.

43. Hu S, Bansal P, Lynch D, Rojas Hernandez CM, Dayao Z. Rituximab, etoposide, methylprednisolone, high-dose cytarabine, and cisplatin in the treatment of secondary hemophagocytic lymphohistiocytosis with classical Hodgkin lymphoma: a case report and review of the literature. *J Med Case Rep* 2016 Dec 20; **10**(1)**:** 365.

44. Hussain S, Hallam S, Beltran L, Haroon A, Majumdar K, Shamash J*, et al.* Intravascular large B-cell lymphoma presenting as a pituitary mass with bilateral adrenal enlargement and haemophagocytic lymphohistiocytosis. *Br J Haematol* 2018 Jun; **181**(6)**:** 851-852.

45. Inagaki N, Sugimoto K, Hosone M, Isobe Y, Yamamoto Y, Sasaki M*, et al.* Disseminated Mucor infection and thrombotic microangiopathy in lymphoma-

associated hemophagocytic syndrome. *International journal of hematology* 2008 Oct; **88**(3)**:** 355-356.

46. Isotani H, Kameoka K. Hemophagocytic syndrome associated with B cell lymphoma in a patient with mitochondrial diabetes. *Ann Hematol* 2001 Mar; **80**(3)**:** 187-188.

47. Jamil A, Nadzri N, Harun N, Ong CL. Primary cutaneous diffuse large B-cell lymphoma leg type presenting with hemophagocytic syndrome. *J Am Acad Dermatol* 2012 Nov; **67**(5)**:** e222-223.

48. Jang KA, Choi JH, Sung KJ, Moon KC, Koh JK, Kwon YM*, et al.* Primary CD56 + nasal-type T/natural killer-cell subcutaneous panniculitic lymphoma: presentation as haemophagocytic syndrome. *The British journal of dermatology* 1999 Oct; **141**(4)**:** 706-709.

49. Jassal DS, Kasper K, Morales C, Rubinger M. Autologous peripheral stem cell transplantation for aggressive hemophagocytic syndrome associated with T-cell lymphoma: case study and review. *Am J Hematol* 2002 Jan; **69**(1)**:** 64-66.

50. Jia J, Song Y, Lin N, Liu W, Ping L, Zheng W*, et al.* Clinical features and survival of extranodal natural killer/T cell lymphoma with and without hemophagocytic syndrome. *Ann Hematol* 2016 Dec; **95**(12)**:** 2023-2031.

51. Jiang T, Ding X, Lu W. The Prognostic Significance of Beta2 Microglobulin in Patients with Hemophagocytic Lymphohistiocytosis. *Dis Markers* 2016; **2016:** 1523959.

52. Jung B, Zoric L, Chanques G, Konate A, Nocca D, Jaber S. Acute abdomen and severe lactic acidosis can lead to a surprising diagnosis. *Intensive Care Med* 2010 Jan; **36**(1)**:** 169-170.

53. Karkouche R, Ingen-Housz-Oro S, Le Gouvello S, Charlotte F, Thomas M, Zehou O*, et al.* Primary cutaneous aggressive epidermotropic CD8+ T-cell lymphoma with KIR3DL2 and NKp46 expression in a human immunodeficiency virus carrier. *J Cutan Pathol* 2015 Mar; **42**(3)**:** 199-205.

54. Kato T, Tanabe J, Kanemoto M, Kobayashi C, Morita S, Karahashi T. A case of extranodal NK/T-cell lymphoma, nasal type mimicking typical manifestations of adult-onset Still's disease (AOSD) with hemophagocytic syndrome: diagnostic consideration between malignant lymphoma without lymphadenopathy and AOSD. *Modern rheumatology* 2009; **19**(6)**:** 675-680.

55. Kim JE, Kim CW, Park SH, Chi JG. Hemophagocytic syndrome associated with occult B-cell lymphoma: an autopsy case. *J Korean Med Sci* 1998 Feb; **13**(1)**:** 77-80.

56. Kim MS, Cho YU, Jang S, Seo EJ, Lee JH, Park CJ. A Case of Primary Bone Marrow Diffuse Large B-cell Lymphoma Presenting With Fibrillar Projections and Hemophagocytic Lymphohistiocytosis. *Ann Lab Med* 2017 Nov; **37**(6)**:** 544-546.

57. Kobayashi T, Ohno H. Intravascular large B-cell lymphoma associated with t(14;19)(q32;q13) translocation. *Intern Med* 2011; **50**(18)**:** 2007-2010.

58. Koduri PR, Carandang G, DeMarais P, Patel AR. Hyperferritinemia in reactive hemophagocytic syndrome report of four adult cases. *Am J Hematol* 1995 Jul; **49**(3)**:** 247-249.

59. Kojima H, Takei N, Mukai Y, Hasegawa Y, Suzukawa K, Nagata M*, et al.* Hemophagocytic syndrome as the primary clinical symptom of Hodgkin's disease. *Ann Hematol* 2003 Jan; **82**(1)**:** 53-56.

60. Krishna R, Byrne E, Burbridge J, Salooja N, Naresh KN. The Hammersmith hospital hematopathology case of the month: hemophagocytic lymphohistiocytosis secondary to Epstein-Barr virus associated T-cell lymphoma. *Leuk Lymphoma* 2011 Jun; **52**(6)**:** 1127-1132.

61. Kueck BD, Hanson CA, Weissman DE, Bayliss K. Primary lymph node presentation of angiocentric lymphoma associated with features of a hemophagocytic syndrome. *Am J Hematol* 1989 Feb; **30**(2)**:** 104-111.

62. Kuo CY, Yeh ST, Huang CT, Lin SF. Diffuse large B-cell lymphoma presenting with type B lactic acidosis and hemophagocytic syndrome. *Kaohsiung J Med Sci* 2014 Aug; **30**(8)**:** 428-429.

63. Kwon SY, Lee JJ, Chung IJ, Kim HJ, Park MR, Kim HS*, et al.* Hepatosplenic B-cell lymphoma associated with hemophagocytic syndrome: a case report. *J Korean Med Sci* 1999 Dec; **14**(6)**:** 671-674.

64. Lecronier M, Prendki V, Gerin M, Schneerson M, Renvoise A, Larroche C*, et al.* Q fever and Mediterranean spotted fever associated with hemophagocytic syndrome: case study and literature review. *Int J Infect Dis* 2013 Aug; **17**(8)**:** e629-633.

65. Lee SY, Wu CW, Chang WH, Ku HC. Fever and jaundice caused by hemophagocytic syndrome. *J Formos Med Assoc* 2019 Feb; **118**(2)**:** 649-650.

66. Lin TA, Yang CF, Liu YC, Liu JH, Chiou TJ, Hsiao LT*, et al.* Hematopoietic stem cell transplantation for subcutaneous panniculitis-like T-cell lymphoma: single center experience in an Asian population. *International journal of hematology* 2019 Feb; **109**(2)**:** 187-196.

67. Ma L, Katz Y, Sharan KP, Schwarting R, Kim AS. Epstein-Barr virus positive anaplastic large cell lymphoma: myth or reality? *Int J Clin Exp Pathol* 2010 Nov 20; **4**(1)**:** 100-110.

68. Machaczka M, Nahi H, Karbach H, Klimkowska M, Hagglund H. Successful treatment of recurrent malignancy-associated hemophagocytic lymphohistiocytosis with a modified HLH-94 immunochemotherapy and allogeneic stem cell transplantation. *Med Oncol* 2012 Jun; **29**(2)**:** 1231-1236.

69. Machaczka M, Vaktnas J. Haemophagocytic syndrome associated with Hodgkin lymphoma and Pneumocystis jiroveci pneumonitis. *Br J Haematol* 2007 Sep; **138**(6)**:** 672.

70. Maejima H, Tanei R, Morioka T, Miyakoshi S. Haemophagocytosis-related intravascular large B-cell lymphoma associated with skin eruption. *Acta Derm Venereol* 2011 May; **91**(3)**:** 339-340.

71. Matsumura Y, Kuroda J, Shimura Y, Kiyota M, Yamamoto-Sugitani M, Kobayashi T*, et al.* Cyclosporine A and reduced-intensity conditioning allogeneic stem cell transplantation for relapsed angioimmunoblastic T cell lymphoma with hemophagocytic syndrome. *Intern Med* 2012; **51**(19)**:** 2785-2787.

72. Mayson E, Saverimuttu J, Warburton P. Two-faced haemophagocytic lymphohistiocytosis: comparative review of two cases of adult haemophagocytic lymphohistiocytosis. *Intern Med J* 2014 Feb; **44**(2)**:** 198-201.

73. Miura T, Kawakami Y, Sato M, Ohtsuka M, Yamamoto T. Hemophagocytic syndrome occurred in a patient with subcutaneous panniculitis-like T-cell lymphoma without overt skin lesion: successful treatment with steroid pulse therapy. *J Dermatol* 2011 Nov; **38**(11)**:** 1113-1115.

74. Miyahara M, Sano M, Shibata K, Matsuzaki M, Ibaraki K, Shimamoto Y*, et al.* B-cell lymphoma-associated hemophagocytic syndrome: clinicopathological characteristics. *Ann Hematol* 2000 Jul; **79**(7)**:** 378-388.

75. Mizutani S, Kuroda J, Shimura Y, Kobayashi T, Tsutsumi Y, Yamashita M*, et al.* Cyclosporine A for chemotherapy-resistant subcutaneous panniculitis-like T cell lymphoma with hemophagocytic syndrome. *Acta Haematol* 2011; **126**(1)**:** 8-12.

76. Morita Y, Kenzaka T, Yoshimoto H, Ohno N. Hodgkin's lymphoma preceded by haemophagocytic lymphohistiocytosis. *BMJ Case Rep* 2013 Jun 10; **2013**.

77. Motegi S, Nishizaki Y, Muramatsu C, Nakamura H, Kobayashi F, Shiozawa H*, et al.* Hemophagocytic syndrome in ileum-origin B-cell lymphoma. *J Gastroenterol* 2003; **38**(10)**:** 995-999.

78. Murase T, Nakamura S, Tashiro K, Suchi T, Hiraga J, Hayasaki N*, et al.* Malignant histiocytosis-like B-cell lymphoma, a distinct pathologic variant of intravascular lymphomatosis: a report of five cases and review of the literature. *Br J Haematol* 1997 Dec; **99**(3)**:** 656-664.

79. Nakayama S, Morita Y, Espinoza JL, Rai S, Taniguchi Y, Taniguchi T*, et al.* Multiple cytokine-producing aggressive EBV-positive diffuse large B cell lymphoma, not otherwise specified with hemophagocytic syndrome. *Ann Hematol* 2020 Feb; **99**(2)**:** 381-383.

80. Narimatsu H, Morishita Y, Saito S, Shimada K, Ozeki K, Kohno A*, et al.* Usefulness of bone marrow aspiration for definite diagnosis of Asian variant of intravascular lymphoma: four autopsied cases. *Leuk Lymphoma* 2004 Aug; **45**(8)**:** 1611-1616.

81. Neistadt B, Carrubba A, Zaretsky MV. Natural killer/T-cell lymphoma and secondary haemophagocytic lymphohistiocytosis in pregnancy. *BMJ Case Rep* 2018 Sep 15; **2018**.

82. Ng CS, Chan JK, Cheng PN, Szeto SC. Nasal T-cell lymphoma associated with hemophagocytic syndrome. *Cancer* 1986 Jul 1; **58**(1)**:** 67-71.

83. Noguchi M, Kawano Y, Sato N, Oshimi K. T-cell lymphoma of CD3+CD4+CD56+granular lymphocytes with hemophagocytic syndrome. *Leuk Lymphoma* 1997 Jul; **26**(3-4)**:** 349-358.

84. Nosari A, Oreste PL, Biondi A, Costantini MC, Santoleri L, Intropido L*, et al.* Hepato-splenic gammadelta T-cell lymphoma: a rare entity mimicking the hemophagocytic syndrome. *Am J Hematol* 1999 Jan; **60**(1)**:** 61-65.

85. Notaro E, Shustov A, Chen X, Shinohara MM. Kikuchi-Fujimoto Disease Associated With Subcutaneous Panniculitis-Like T-Cell Lymphoma. *Am J Dermatopathol* 2016 Jun; **38**(6)**:** e77-80.

86. Obama K, Tara M, Niina K. L-asparaginase-Based induction therapy for advanced extranodal NK/T-cell lymphoma. *International journal of hematology* 2003 Oct; **78**(3)**:** 248-250.

87. Ohno H, Takimoto K. Gastric mucosa-associated lymphoid tissue lymphoma complicated with hemophagocytic syndrome in an elderly woman. *Ann Hematol* 2010 Nov; **89**(11)**:** 1175-1176.

88. Ohno T, Ueda Y, Nagai K, Takahashi T, Konaka Y, Takamatsu T*, et al.* The serum cytokine profiles of lymphoma-associated hemophagocytic syndrome: a comparative analysis of B-cell and T-cell/natural killer cell lymphomas. *International journal of hematology* 2003 Apr; **77**(3)**:** 286-294.

89. Okamoto M, Yamaguchi H, Isobe Y, Yokose N, Mizuki T, Tajika K*, et al.* Analysis of triglyceride value in the diagnosis and treatment response of secondary hemophagocytic syndrome. *Intern Med* 2009; **48**(10)**:** 775-781.

90. Pasvolsky O, Zoref-Lorenz A, Abadi U, Geiger KR, Hayman L, Vaxman I*, et al.* Hemophagocytic lymphohistiocytosis as a harbinger of aggressive lymphoma: a case series. *International journal of hematology* 2019 May; **109**(5)**:** 553-562.

91. Peeters P, Sennesael J, De Raeve H, De Waele M, Verbeelen D. Hemophagocytic syndrome and T-cell lymphoma after kidney transplantation: a case report. *Transpl Int* 1997; **10**(6)**:** 471-474.

92. Pongpairoj K, Rerknimitr P, Wititsuwannakul J, Asawanonda P. Eruptive telangiectasia in a patient with fever and haemophagocytic syndrome. *Clin Exp Dermatol* 2016 Aug; **41**(6)**:** 696-698.

93. Real E, Gomez A, Alcaraz MJ, Saez AI, Pastor E, Grau E. Fulminant hemophagocytic syndrome as presenting feature of T-cell lymphoma and Epstein-Barr virus infection. *Haematologica* 2000 Apr; **85**(4)**:** 439-440.

94. Rivera XI, McGhan LJ, Schatz JH, Puvvada SD. Double hit lymphoma presenting as haemophagocytic lymphohistiocytosis. *BMJ Case Rep* 2017 May 15; **2017**.

95. Romero Fernandez E, Pardo JR, Doyle A, Albendea MC, de la Rua AR. Hemophagocytic syndrome associated NK/T nasal type lymphoma presenting as hypereosinophilic syndrome: a case report and literature review. *Leuk Res* 2011 Jul; **35**(7)**:** e97-99.

96. Rutnin S, Porntharukcharoen S, Boonsakan P. Clinicopathologic, immunophenotypic, and molecular analysis of subcutaneous panniculitis-like T-cell lymphoma: A retrospective study in a tertiary care center. *J Cutan Pathol* 2019 Jan; **46**(1)**:** 44-51.

97. Sano H, Kobayashi R, Tanaka J, Hashino S, Ota S, Torimoto Y*, et al.* Risk factor analysis of non-Hodgkin lymphoma-associated haemophagocytic syndromes: a multicentre study. *Br J Haematol* 2014 Jun; **165**(6)**:** 786-792.

98. Sano T, Sakai H, Takimoto K, Ohno H. Rituximab alone was effective for the treatment of a diffuse large B-cell lymphoma associated with hemophagocytic syndrome. *Int J Clin Oncol* 2007 Feb; **12**(1)**:** 59-62.

99. Sasaki K, Yamato M, Yasuda K, Rakugi H, Isaka Y. Rhabdomyolysis caused by peripheral T-cell lymphoma in skeletal muscle. *Am J Emerg Med* 2013 Oct; **31**(10)**:** 1537 e1533-1535.

100. Sato T, Kogawa K, Iyama S, Kobayashi D, Sato Y, Kuribayashi K*, et al.* Successful treatment of advanced peripheral T-cell lymphoma with an angiocentric growth pattern complicated with hemophagocytic syndrome by high-dose chemotherapy and autologous peripheral blood stem cell transplantation. *Ann Hematol* 2002 Dec; **81**(12)**:** 739-743.

101. Shimazaki C, Inaba T, Shimura K, Okamoto A, Takahashi R, Hirai H*, et al.* B-cell lymphoma associated with haemophagocytic syndrome: a clinical, immunological and cytogenetic study. *Br J Haematol* 1999 Mar; **104**(4)**:** 672-679.

102. Shimazaki C, Inaba T, Okano A, Hatsuse M, Takahashi R, Hirai H*, et al.* Clinical characteristics of B-cell lymphoma-associated hemophagocytic syndrome (B-LAHS): comparison of CD5+ with CD5- B-LAHS. *Intern Med* 2001 Sep; **40**(9)**:** 878-882.

103. Shimizu I, Ichikawa N, Yotsumoto M, Sumi M, Ueno M, Kobayashi H. Asian variant of intravascular lymphoma: aspects of diagnosis and the role of rituximab. *Intern Med* 2007; **46**(17)**:** 1381-1386.

104. Shimizu Y, Tanae K, Takahashi N, Kohri M, Arai E, Bessho M*, et al.* Primary cutaneous anaplastic large-cell lymphoma presenting with hemophagocytic syndrome: a case report and review of the literature. *Leuk Res* 2010 Feb; **34**(2)**:** 263-266.

105. Soiza RL, Ghosh S, McAlpine JK, Vickers MA. Non-fatal haemophagocytic syndrome in an elderly patient. *Age Ageing* 2005 Sep; **34**(5)**:** 522-524.

106. Suvorava N, Richmond S, Patel N, Bell B, Mesa H. Between a rock and a hard place. *Am J Hematol* 2016 Mar; **91**(3)**:** 351-353.

107. Tabata R, Tabata C, Kimura T, Nagai T, Yasumizu R. Prominent granulomas in bone marrow in disseminated lymphoma with hemophagocytic syndrome. *Ann Hematol* 2011 Nov; **90**(11)**:** 1365-1367.

108. Takahashi E, Kajimoto K, Fukatsu T, Yoshida M, Eimoto T, Nakamura S. Intravascular large T-cell lymphoma: a case report of CD30-positive and ALK-negative anaplastic type with cytotoxic molecule expression. *Virchows Arch* 2005 Dec; **447**(6)**:** 1000-1006.

109. Takahashi N, Miura I, Chubachi A, Miura AB, Nakamura S. A clinicopathological study of 20 patients with T/natural killer (NK)-cell lymphoma-associated hemophagocytic syndrome with special reference to nasal and nasal-type NK/T-cell lymphoma. *International journal of hematology* 2001 Oct; **74**(3)**:** 303-308.

110. Takahashi T, Kanda Y, Mori M, Saito T, Chiba S, Mitani K*, et al.* B cell lymphoma-associated hemophagocytic syndrome after PBSCT. *Bone Marrow Transplant* 1998 Mar; **21**(6)**:** 623-625.

111. Takami A, Nakao S, Ueda M, Miura Y, Matsuda T, Kawamura Y. Successful treatment of B-cell lymphoma associated with hemophagocytic syndrome using autologous peripheral blood CD34 positive cell transplantation followed by induction of autologous graft-versus-host disease. *Ann Hematol* 2000 Jul; **79**(7)**:** 389-391.

112. Tan B, Abdelmalek C, O'Donnell JE, Toltaku T, Chaudhry R, Wang JC*, et al.* A Case Report of Primary Nasal Natural Killer (NK)/T-Cell Lymphoma in an African American Patient Presenting with Hemophagocytic Syndrome. *Am J Case Rep* 2017 Feb 14; **18:** 160-165.

113. Tatara R, Sato M, Fujiwara S, Oh I, Muroi K, Ozawa K*, et al.* Hemoperfusion for Hodgkin lymphoma-associated hemophagocytic lymphohistiocytosis. *Intern Med* 2014; **53**(20)**:** 2365-2368.

114. Terrier B, Aouba A, Vasiliu V, Charlier C, Delarue R, Buzyn A*, et al.* Intravascular lymphoma associated with haemophagocytic syndrome: a very rare entity in western countries. *Eur J Haematol* 2005 Oct; **75**(4)**:** 341-345.

115. Tsai AS, Ko CW, Yeh HZ, Chang CS, Wang RC. Peripheral T-cell lymphoma of the colon associated with hemophagocytic lymphohistiocytosis. *J Chin Med Assoc* 2013 Mar; **76**(3)**:** 169-172.

116. Tsuji T, Hirano T, Yamasaki H, Tsuji M, Tsuda H. A high sIL-2R/ferritin ratio is a useful marker for the diagnosis of lymphoma-associated hemophagocytic syndrome. *Ann Hematol* 2014 May; **93**(5)**:** 821-826.

117. Tsukamoto Y, Katsunobu Y, Omura Y, Maeda I, Hirai M, Teshima H*, et al.* Subcutaneous panniculitis-like T-cell lymphoma: successful initial treatment with prednisolone and cyclosporin A. *Intern Med* 2006; **45**(1)**:** 21-24.

118. Tzeng HE, Teng CL, Yang Y, Young JH, Chou G. Occult subcutaneous panniculitis-like T-cell lymphoma with initial presentations of cellulitis-like skin lesion and fulminant hemophagocytosis. *J Formos Med Assoc* 2007 Feb; **106**(2 Suppl)**:** S55-59.

119. Uehara T, Yokota A, Onoda M, Yamamoto K, Terano T. Successful autologous peripheral blood stem cell transplantation for a patient with primary adrenal lymphoma with hemophagocytic syndrome. *Clin Lymphoma Myeloma* 2008 Jun; **8**(3)**:** 184-187.

120. Uni M, Yoshimi A, Maki H, Maeda D, Nakazaki K, Nakamura F*, et al.* Successful treatment with recombinant thrombomodulin for B-cell lymphoma-associated hemophagocytic syndrome complicated by disseminated intravascular coagulation. *Int J Clin Exp Pathol* 2013; **6**(6)**:** 1190-1194.

121. Varghese D, Haseer Koya H, Cherian SV, Mead K, Sharma A, Sharma N*, et al.* Hemophagocytic lymphohistiocytosis: an uncommon presentation of enteropathy-associated T-cell lymphoma. *J Clin Oncol* 2013 May 1; **31**(13)**:** e226-230.

122. Wang J, Ding W, Gao L, Yao W, Chen M, Zhao S*, et al.* High Frequency of Bone Marrow Involvement in Intravascular Large B-Cell Lymphoma. *Int J Surg Pathol* 2017 Apr; **25**(2)**:** 118-126.

123. Wang Y, Huang W, Hu L, Cen X, Li L, Wang J*, et al.* Multicenter study of combination DEP regimen as a salvage therapy for adult refractory hemophagocytic lymphohistiocytosis. *Blood* 2015 Nov 5; **126**(19)**:** 2186-2192.

124. Watabe R, Shibata K, Hirase N, Kodera T, Muta K, Nishimura J*, et al.* Angiotropic B-cell lymphoma with hemophagocytic syndrome associated with syndrome of inappropriate secretion of antidiuretic hormone. *Ann Hematol* 2000 Oct; **79**(10)**:** 581-584.

125. Xu Z, Burns BF. Hemophagocytosis due to bone marrow ALCL, ALK. *Blood* 2014 Jul 24; **124**(4)**:** 478.

126. Yamamoto K, Nagata K, Hamaguchi H. Translocation (11;14)(q13;q32) in CD5-positive B-cell lymphoma associated with haemophagocytic syndrome. *Br J Haematol* 1999 Sep; **106**(4)**:** 1069-1070.

127. Yamamoto K, Nakamura Y, Arai H, Aoyagi M, Saito K, Furusawa S*, et al.* Translocation (14;19)(q32;q13) detected by spectral karyotyping and lack of BCL3 rearrangement in CD5-positive B-cell lymphoma associated with hemophagocytic syndrome. *Cancer Genet Cytogenet* 2001 Oct 1; **130**(1)**:** 38-41.

128. Yu JT, Hwang WL, Wang RC, Teng CL. Reduced intensity conditioning allogeneic hematopoietic stem cell transplant could be beneficial to angioimmunoblastic T-cell lymphoma patients with hemophagocytic lymphohistiocytosis. *Ann Hematol* 2012 May; **91**(5)**:** 805-807.

129. Yu JT, Wang CY, Yang Y, Wang RC, Chang KH, Hwang WL*, et al.* Lymphoma-associated hemophagocytic lymphohistiocytosis: experience in adults from a single institution. *Ann Hematol* 2013 Nov; **92**(11)**:** 1529-1536.

130. Yun S, Taverna JA, Puvvada SD, Anwer F. NK/T-cell non-Hodgkin's lymphoma with secondary haemophagocytic lymphohistiocytosis treated with matched unrelated donor allogeneic stem cell transplant. *BMJ Case Rep* 2014 Aug 14; **2014**.

131. Zabernigg A, Fend F, Thaler J, Gattringer C. An unusual case of a splenic gamma/delta T-cell lymphoma with angiocentric tendency and haemophagocytic syndrome. *Leuk Lymphoma* 1996 Nov; **23**(5-6)**:** 631-634.

132. Zhan Y, Teruya-Feldstein J. Immune dysregulation: EBV(+) DLBCL and HLH in a patient with T-LGL. *Blood* 2019 Apr 11; **133**(15)**:** 1695.
